# Entropy generation optimization for the electroosmotic MHD fluid flow over the curved stenosis artery in the presence of thrombosis

**DOI:** 10.1038/s41598-023-42540-0

**Published:** 2023-09-18

**Authors:** Bhupendra K. Sharma, Umesh Khanduri, Nidhish K. Mishra, Ibrahim Albaijan, Laura M. Pérez

**Affiliations:** 1 Department of Mathematics, Birla Institute of Technology and Science Pilani, Rajasthan, India; 2 Department of Basic Science, College of Science and Theoretical Studies Saudi Electronic University, Riyadh, Saudi Arabia; 3 Mechanical Engineering Department, College of Engineering at Al Kharj, Prince Sattam Bin Abdulaziz University, Al Kharj, 16273 Saudi Arabia; 4 Departamento de Física, FACI, Universidad de Tarapacá, Casilla 7D, Arica, 1000000 Chile

**Keywords:** Applied mathematics, Mathematics and computing, Biomedical engineering

## Abstract

The present study deals with the entropy generation analysis on the flow of an electrically conductive fluid (Blood) with $\hbox {Al}_2\hbox {O}_3$-suspended nanoparticles through the irregular stenosed artery with thrombosis on the catheter. The fluid flow can be actuated by the interactions of different physical phenomena like electroosmosis, radiation, Joule heating and a uniform radial magnetic field. The analysis of different shapes and sizes of the nanoparticle is considered by taking the Crocine model. The velocity, temperature, and concentration distributions are computed using the Crank–Nicholson method within the framework of the Debye–Huckel linearization approximation. In order to see how blood flow changes in response to different parameters, the velocity contour is calculated. The aluminium oxide nanoparticles employed in this research have several potential uses in biomedicine and biosensing. The surface’s stability, biocompatibility, and reactivity may be enhanced by surface engineering, making the material effective for deoxyribonucleic acid sensing. It may be deduced that the velocity profile reduces as the nanoparticle’s size grows while depicts the reverse trend for the shape size. In a region close to the walls, the entropy profile decreases, while in the region in the middle, it rises as the magnetic field parameter rises. The present endeavour can be beneficial in biomedical sciences in designing better biomedical devices and gaining insight into the hemodynamic flow for treatment modalities.

## Introduction

Cardiovascular disease (CVD) is a major public health concern due to its high morbidity and mortality rates^1^. This rates upshots in both developed and developing countries due to obesity and poor lifestyle. CVD encompasses a wide range of disorders, including cardiac muscle and vascular system diseases. It is widely recognised that arterial pathologies arise due to the degradation mechanisms involving cholesterol, lipoproteins, and diverse chemical components. These processes predominantly occur at the curvatures or bifurcation points of the arterial wall. Many researchers pointed out that hemodynamic factors play a significant role in the formation and progression of these diseases. Walsh^2^ explained that stenosis and thrombosis result from vascular injury and inflammation. The clot develops due to internal damage to the arterial lumen, and additional clot formation results in stenosis or emboli. The shear stress and hemodynamic parameters affecting stenosis and thrombosis were investigated by Strony et al.^3^. They found that the shape of stenosis had a significant impact on platelet activation and thrombosis development in a diseased artery. Elaqneeb et al.^4^ developed a mathematical model of the stenotic artery with a thrombosis. When they considered the copper nanoparticle, they concluded that an increase in the nanoparticle volume fraction led to a boost in velocity. Tanveer et al.^5^ investigated the MHD (magneto hydrodynamics) Jeffery nanofluid in curved channel with convective boundary conditions. Ahmed and Nadeem^6^ analysed the shape effect of copper nanoparticles through curved stenosed artery. Ahmed and Nadeem^7^ constructed the mathematical model to analyse the effect of MHD on a micropolar fluid flow through 6-types of different stenosis. The study conducted by Shahzadi and Kousar^8^ focused on the development of a mathematical model for analysing the behaviour of a bifurcated stenosed artery, with particular emphasis on incorporating slip effects into the model. The research findings indicated that the angle of bifurcation played a significant role in improving the distribution of shear stress within the main artery. Conversely, the daughter artery exhibited a contrasting pattern, with a decrease in shear stress as the bifurcation angle increased. In their study, Zidan et al.^9^ undertook an investigation aimed at evaluating the entropy generation occurring within a catheterised stenosed artery. The analysis conducted by the researchers revealed that an enhancement in the stenosis height resulted in a heightened intensity of the shear stress profile at the arterial wall. Khanduri and Sharma^10^ conducted a study to analyse the impact of nanoparticle shape, explicitly focusing on hybrid nanoparticles (Au/GO), on blood circulation within a compromised artery. The study incorporated considerations of the Hall and ion effect. The affected artery displayed stenosis along its arterial walls and thrombosis at the central region of the catheter. The researchers concluded that an augmentation in the shape of nanoparticles improved the temperature profile.

Young^11^ analyzed the deposition of plaque along the lumen of the artery disturbed the blood flow and led to mechanical processes advancing in intimal cell proliferation. Flow separation is the main factor in the development of vascular disorders, suggested by Mustapha et al.^12^ They analyzed the unsteady MHD fluid flow through an irregular multi-stenosed arteries and concluded that the flow separation zone shrank with increasing the Hartmann number value. Changdar et al.^13^ discussed the nanoparticle application as drug delivery in the blood flow through an irregular stenosed artery by considering single- and discrete-phase models. Gandhi et al.^14^ discussed magnetic hybrid nanoparticle (Au-$\hbox {Al}_2\hbox {O}_3$/blood) based drug delivery through a bell-shaped occluded artery with joule heating, viscous dissipation and variable viscosity. The application of blood with the applied magnetic field has extensive applications in the biomedical and engineering fields. Kolin^15^ first introduced the concept of MHD in the medical field. The experimental results indicate that when a conducting fluid, such as human blood, is exposed to a magnetic field strength of 10 T, it experiences a retarding force that leads to a $30\%$ decrease in flow. Moreover, the application of an external electric field results in the emergence of an electro-osmotic force, which in turn causes the migration of an electrolyte within a specific conduit. When the conduit is placed in an electrolytic medium, it induces an electrostatic response in which positively charged particles are drawn towards its surface. In contrast, negatively charged ions are pushed away. The Electric Double Layer (EDL) formation occurs due to this phenomenon. Initially, Woodson and Melcher^16^ investigated electrically charged fluid dynamics, also known as electrokinetics or Electrohydrodynamics (EHD). The focus of their study lies in the examination of the behaviour and relationships between ionised particles and the surrounding fluids. Additionally, they investigate the mechanisms that facilitate the movement of fluids, including electrostatics, electrophoresis, and electro-osmosis, among other related phenomena. Whitehead and Rice^17^ applied the Debye–Huckel approximation for the electrokinetic flow through narrow capillaries. In a recent study, Nooren et al.^18^ looked at how Joule heating and various zeta functions affected MHD nanofluid in a microchannel. Their study revealed that increases in the zeta potential retard fluid motion, an essential medical phenomenon, regulating blood flow. This retarding nature occurs due to the presence of impregnable EDL.

The study by Akram et al.^19^ aimed to investigate the electroosmosis impact by comparing the modified Buongiorno and Tiwari-Das model. The investigation demonstrated that the modified Buongiorno model exhibits superior performance as a viscosity model compared to the Tiwari-Das model. In a subsequent study, Abdelsalam et al.^20^ investigated the hemodynamic characteristics of nanofluid flow in a diseased artery affected by both stenosis and aneurysm. The study also considered the influence of electroosmotic forces and the size of the nanoparticles. Shahzadi et al.^21^ conducted a study that aimed to examine the impact of electroosmotic force on the oblique stenosed aneurysmal artery. The researchers utilised a fractional model based on second-grade principles, incorporating ternary nano particles. They placed particular emphasis on the potential advantages of their study in augmenting drug transportation.

Blood is a very complex and marvellous fluid that nurtures life. Over the past few decades, scientists and researchers have been studying to uncover the perplexing behaviour of blood. It is essential to know the behaviour of blood to deal with the pathological conditions faced by animals and human beings. Examining fluid dynamics in a curved conduit is significant in biomedicine due to its ability to closely replicate the complex flow patterns observed in arterial blood vessels. These investigations are of great value in managing patients with coronary pathologies. In the study by Mekheimer et al.^22^, an examination was carried out to analyse the hemodynamic properties of fluid flow in a curved artery, specifically in the context of catheterisation. The researchers’ study clarified that narrower arteries exhibit higher fluidic resistance than wider arteries. Additionally, they found that the velocity profile in non-curved arteries is more significant than that observed in curved arteries. Zaman et al.^23^ examined the effect of different types of nanoparticles through curved stenosed channels. Their study exhibited that the curvature parameter influences the velocity profile, and the symmetric patterns reduce for a higher value of the curvature parameter. Sharma et al.^24^ studied the MHD blood flow through a curved artery by considering the effect of heat transfer and body acceleration. Several other researchers^25,26^ scrutinized the blood flow through the curved stenosis artery. Majorly, researchers considered the blood viscosity a constant, but in reality, it gets influenced by different factors like pressure, temperature and flow rate. Lih et al.^27^ examined that blood viscosity at the low-shear region vary according to hematocrit and blood vessel diameter. Mishra et al.^28^ introduced a mathematical framework to comprehend blood circulation dynamics from the parent artery to the capillary network, considering different entry angles. The primary focus of the investigation revolved around the phenomenon of hematocrit reduction via plasma skimming and the mass flux occurring within the capillary system. The study’s results highlighted that the lowest quantity of red blood cells passing from the main artery to the smaller capillary is observed when both vessels are positioned at a right angle. The variable viscosity is essential whenever blood through a tube or channel is studied. Keeping this aspect in mind, several researchers^29–32^ conducted investigations pertaining to variable viscosity within their model. Baskurt et al.^33^ emphasized the variation in blood viscosity are influenced by hematocrit, RBCs (red blood cells) aggregation, shear stress and mechanical properties of RBCs. Ponalagusamy^34^ developed the mathematical model of the two-fluid model in tapered arterial stenosis. They considered micropolar fluid in the core region and Newtonian fluid in the peripheral plasma region with variable viscosity. Sharma and Kumawat^35^ temperature dependent viscosity and thermal conductivity on mhd blood flow through a stretching surface with ohmic effect and chemical reaction. Sharma et al.^36,37^ discussed the impact of viscosity and radiation on the MHD fluid flow thorough a stretching sheet.

The importance of nanoparticles in the field of biomedicine has been emphasised by a combination of theoretical studies and empirical data. The studies shows the importance of nanoparticles to enhance the administration of diagnostic and therapeutic substances. Thus, numerous investigations have been conducted to explore molecular-level functionalities of nanoparticles in the field of life sciences. Shahzadi and Nadeem^38^ conducted a series of studies to investigate the simulation of metallic nanoparticles located within eccentric annuli, while being subjected to the effects of a radial magnetic field. Moreover, a comparative analysis of copper nanoparticles was conducted in a separate study^39^. The study specifically examined the slip effect in oblique cylinders. Furthermore, Shahzadi et al.^40^ conducted a study to examine the influence of different shapes of Ag nanoparticles, including platelets, bricks, and cylinders, within a curved artery. The results indicated that there was an increase in the velocity field as the curvature parameter was raised. In their study, Kumar et al.^41^ performed an investigation on the features of flow and heat transfer within a porous medium, specifically focusing on the application of various hybrid nanofluids. On the other hand, the study conducted by Imran et al.^42^ centred on the analysis of the flow of an incompressible Jeffrey nanofluid through a vertical tube. The results of their study revealed a positive relationship between velocity and nanoparticle concentration with thermophysical parameters, while temperature showed a negative association. Jamil et al.^43^, employed Caputo-Fabrizio fractional derivatives to examine the flow characteristics of Casson fluid within a constricted artery. The researchers observed that an increase in the Hartmann number resulted in an elevation of the concentration of magnetic particles, consequently leading to an augmentation of fluid viscosity and a decrease in fluid velocity. Hassan et al.^44^ conducted an independent study to examine the characteristics of the boundary layer flow of nanofluid over a movable wedge. A decrease in the velocity field was observed as the nanoparticle volume fraction increased. Furthermore, several other researchers^45–49^ have conducted research on the topic of nanofluid flow through curved channels, thereby making valuable contributions to the expanding scholarly discourse in this field.

In the biological system, metabolism is the central process, providing the energy needed to sustain life. The heat transfer and energy losses are incurred in this process causing the disorders (entropy). The entropy generation is associated with the thermodynamic irreversibility process that is associated with the second law of thermodynamics. The mitigation or reduction of the energy losses is desirable and one of the focus area in bio-inspired engineering system. The entropy generation is classified in two physical framework: reversible and irreversible process. The reversible process are those where change of entropy is zero and non-zero change in entropy signifies the irreversible process. Although, all the processes that occurs in nature are irreversible. Several factors associated in the biological process for production of entropy such as (viscosity) fluid friction, exposure to radiation and magnetic field (associated with iron particle present in hemoglobin molecule), electric field (associated with ions), etc. Bejan^50^ pioneered the entropy analysis by studying the four fundamental way of heat conductive process. According to their study the thermal efficiency of the system can be optimized by reducing the overall entropy. Moreover, they concluded that the viscous dissipation and heat transfer were the crucial one for entropy generation in the system. A very few study^51–53^ has been conducted in the field of biological systems. Aoki et al.^54^ investigated the human body’s entropy production at the basal conditions and calculated using the energetic data obtained from the respiration calorimeter. They determined that the impacts of the forced air current and clothes did not influence entropy creation. It’s conceivable that there are physiological systems that can keep the body’s entropy production at constant levels. For the analysis of entropy production utilising a ferromagnetic nanofluid, Akbar and Butt^55^ employed the mathematical model of composite stenosis arteries with permeable walls. Gandhi et al.^56^ took into account the various nanoparticle shapes effect on the multi-stenosed artery exposed to heat radiation to conduct their entropy study. Further theoretical investigation of the MHD two-phase across a permeable curved artery with varying viscosity and radiation was reported by Kumawat et al.^57^. They found that arterial wall permeability and curvature are the most critical risk factors for atherosclerosis.

Inspired by the aforementioned studies, the present research endeavours to investigate a previously unexplored domain, specifically examining the combined effects of nanoparticles’ shape and size, alongside Joule heating, electroosmosis, radial magnetic fields, and radiation, on the blood flow dynamics within a curved stenosed artery with thrombosis. To fill this void in the existing research, we examined the flow of blood containing suspended $\hbox {Al}_2\hbox {O}_3$ nanoparticles through irregular stenosis while also considering the presence of thrombosis on the catheter walls. The nanoparticles under consideration are categorised as porous metallic oxides, known for their significant surface areas and impressive resistance to chemical and mechanical disturbances. The extensive accessibility of these nanoscale entities makes them economically feasible for incorporation into diverse biomedical applications.

This study examines the impact of a uniform radial magnetic field, electroosmosis , and radiation on a system. The hematocrit dependent viscosity model is taken into the consideration. In this study, we have chosen to adopt a curvilinear coordinate system along with mild stenosis assumptions to reduced the complexity of the governing equations. These governing equations are discretized using the Crank–Nicolson method and further solved in the MatLab under the appropriate boundary conditions.

The salient contributions of this research are as follows:

- Investigation of the impact of nanoparticle shape and size on the flow behavior within a curved artery.
- To investigate the impact of variable viscosity on the flow dynamics within a stenosed artery with thrombosis at the centre of the catheter wall, specifically by considering the hematocrit-dependent viscosity model.
- Entropy generation analyzation on the diseased artery by considering the combined effects of Joule heating, electro-osmosis, radial magnetic field and radiation.

## Mathematical formulation

A study is undertaken to examine the hemodynamics of blood flow in a pathological arterial segment that exhibits irregular stenoses and thrombosis at the central region of the catheterised tube. The flow is characterised by being unsteady, laminar, incompressible and fully developed, exhibiting an axisymmetric configuration. To enhance the analysis, a curvilinear coordinate system is employed, where the radial and axial coordinates are represented as $r_1$ and $z_1$ respectively. The adoption of axisymmetry enables the elimination of any dependence on the variable $\theta$ in the flow. The assumption is made that the induced magnetic field is very small, as it is considered insignificant in comparison with an applied magnetic field. As a component of the research, the introduction of aluminium oxide nanoparticles into the bloodstream is conducted to investigate their impact on the flow dynamics as they pass through the afflicted arterial vessel.

### Geometrical representation of the model

**Figure 1:**
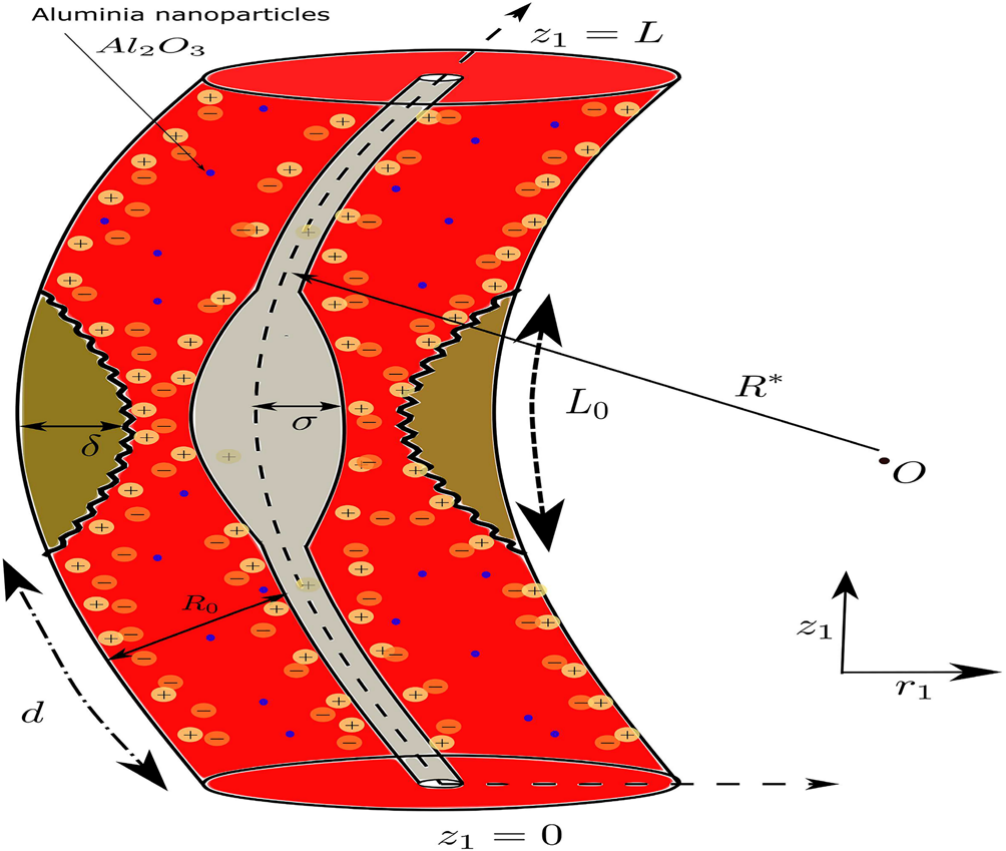
Physical sketch of the irregular-shaped constricted artery with $\hbox {Al}_2\hbox {O}_3$ nanoparticles.

The visual representation of the affected arterial structure is depicted in Fig. 1. The depiction of the arterial configuration involves the use of two concentric tubes, where the radius is represented as $R^{'}$, originating from the central point *O*. The geometric characterization pertains to an irregularly shaped stenotic condition is given as follows^56,58^:

1 $$\begin{aligned} \eta (\tilde{z}_1) = {\left\{ \begin{array}{ll} R_0-2\delta \left[ \cos (\frac{2 \pi }{L_0}(\frac{\tilde{z}_1-\tilde{d}}{2}-\frac{L_0}{4}) -\frac{7}{100} \cos (\frac{32 \pi }{L_0}(\tilde{z}_1-\tilde{d}-\frac{L_0}{2})) \right] &{} \tilde{d}\le \tilde{z}_1\le \tilde{d}+L_0, \\ R_0 &{} \text {otherwise }, \end{array}\right. } \end{aligned}$$

Let $\eta$ denote the radius of the stenosis segment, which possesses a length denoted by $L_0$. Additionally, *d* represents the distance of the stenosis segment from the initial position *P*. The geometric characteristics of the clot are described as follows:

2 $$\begin{aligned} \epsilon (\tilde{z}_1) = {\left\{ \begin{array}{ll} R_0(c+ \sigma \exp (-\frac{\pi ^2}{L_0}(\tilde{z}_1-\tilde{z}_d-0.5L_0)^2)), &{} \tilde{d}<\tilde{z}_1 \le \tilde{d}+\frac{3}{2}L_0, \\ cR_0, &{} \text {otherwise}, \end{array}\right. } \end{aligned}$$

where $cR_0$ denotes the radius of the inner tube, or catheter, wherein the parameter *c* is significantly smaller than unity ($c<< 1$). The clot axial displacement , with its utmost elevation denoted by $\sigma$, is represented by the variable $z_d$.

### Mathematical formulation

#### Electrohydrodynamics (EHD)

Blood encompasses various ionic constituents like atoms or molecules that gain or lose electrons and thus carry an electric charge, which confers the properties of an electrically conducting fluid upon it. In consideration of this, we investigated the effects of introducing an electric field $(0,0,E_0)$ and an external magnetic field ${\textbf {B}}=(\frac{ \tilde{R}^* B_0}{\tilde{r}_1+\tilde{R}^*},0,0)$ subjected to the blood flow in the afflicted arterial, where $B_0$ remains constant. The current density and Lorentz force is given as^59^:

3 $$\begin{aligned} \frac{{\textbf {J}}.{\textbf {J}}}{\sigma _{nf}} =&\sigma _{nf}\left( \frac{ \tilde{R}^* B_0}{\tilde{r}_1+\tilde{R}^*}\right) ^2 (\tilde{w}_1 ^2) + \sigma _{nf}E_0^2 , \end{aligned}$$

4 $$\begin{aligned} {\textbf {J}} \times {\textbf {B}} =&\left( 0,\sigma _{nf}\left( \frac{ \tilde{R}^* }{\tilde{r}+\tilde{R}^*}\right) B_0E_0,-\sigma _{nf}\left( \frac{ \tilde{R}^* B_0}{\tilde{r}_1+\tilde{R}^*}\right) ^2 \tilde{w}_1 \right) , \end{aligned}$$

where $\sigma _{nf}$ and *J* signifies the electric conductivity and current density vector , respectively. The phenomenon of electroosmosis occurs in the present case as the solid conduct, such as an arterial walls or a catheter, interfaces with an electrolyte solution, such as blood. The occurrence of this interaction results in the formation of an electrical double layer (EDL) in the proximity of the solid surface as a consequence of disparities in ion concentrations. The mathematical representation of the electroosmotic potential is given by the Poisson–Boltzmann equation as^60,61^:

5 $$\begin{aligned} \nabla ^2 \tilde{\Phi } =-\frac{\rho _e}{\epsilon }, \end{aligned}$$

where $\psi$ denotes the electro-osmotic function, while $\epsilon$ represents the dielectric constant. The variable $\rho _e$ is explicitly expressed as follows:

6 $$\begin{aligned} \rho _e=(n^{+}-n^{-})e_0z_0. \end{aligned}$$

The number density of cations and anions can be characterized by the Boltzmann distribution which is given as:

7 $$\begin{aligned} n^{\pm } = n_{0} \exp (\pm \frac{ e_0z_0 \tilde{\Phi }}{k_B T_{avg}}), \end{aligned}$$

where $$k_B$$ is Boltzmann constant , $$e_0$$ is electric constant, $$z_0 $$ is the charge balance . Combining Eqs. 6 and 7 and using the Debye–Huckel linearizion, we get:

8 $$\begin{aligned} \rho _e = - \frac{2e_0^2z_0^2n_0}{k_B T_{avg}} \tilde{\Phi } \end{aligned}$$

By using Eqs. 5 and 8 , the Poisson equation takes the form:

9 $$\begin{aligned} \Biggl (\frac{\partial ^2 }{\partial \tilde{r}_1{^2}} + \frac{1}{\tilde{r}_1 +\tilde{R}^*}\frac{\partial }{\partial \tilde{r}_1} + \left( \frac{\tilde{R}^*}{\tilde{r}_1 +\tilde{R}^*} \right) ^2 \frac{\partial ^2 }{\partial \bar{z}_1^2} \Biggr )\tilde{\Phi } = \frac{\tilde{\Phi }}{q_m^2}, \end{aligned}$$

where $$q_m =\frac{1}{e_0z_0} \sqrt{\frac{\epsilon k_B T_{avg}}{2n_0 }}$$.

#### Viscosity model

Corcione^62^ introduced a theoretical framework for investigating the interrelation between the diameters of nanoparticles $(d_p)$ with nanofluid viscosity $\mu _{nf}$.

10 $$\begin{aligned} \frac{\mu _{nf}}{\mu _f}=\frac{1}{1-34.87(\frac{d_p}{d_f})^{-0.3} \phi ^{1.03}}, \end{aligned}$$

where molecular diameter $d_f$ of the base fluid is provided as follows:

11 $$\begin{aligned} d_f= \left[ \frac{6M}{N \pi \rho _{f_0}}\right] ^{\frac{1}{3}}. \end{aligned}$$

N represents the Avogadro constant $(6.022*10^{23})$, applicable to nanoparticles within the size range of 25 to 200 nm and concentrations spanning from $0.01$ to $7.1\%$.

The abundance of suspended entities within the circulatory system is primarily due to erythrocytes, also known as red blood cells or RBCs. These cells significantly impact the biomechanical properties of blood. The variability of blood viscosity is influenced by the spatial arrangement of its particles, which is a crucial factor examined in the subsequent model proposed in this study^63^.

12 $$\begin{aligned} \mu _f =\mu _0[1+ \gamma _{1} h(\tilde{r}_1)], \end{aligned}$$

where $h(\tilde{r}_1)= h_m[1-(\frac{\tilde{r}_1}{R_0})^m]$, $h_m$ signifies the maximum level of hematocrit with $\gamma _{1}$ as constant.

#### Governing equations

Based on the previously mentioned assumption regarding magnetohydrodynamic (MHD) interaction, the governing equations are provided as follows^63,64^:

* Continuity Equation*

13 $$\begin{aligned} \frac{\partial \tilde{u}_1}{\partial \tilde{r}_1} +\frac{ \tilde{u}_1}{ \tilde{r}_1 + \tilde{R}^*}+ \frac{\tilde{R}^*}{\tilde{r}_1 +\tilde{R}^*}\frac{\partial \tilde{w}_1}{\partial \tilde{z}_1}=0. \end{aligned}$$

*Momentum* (in $r_1^*$-direction)

14 $$\begin{aligned} \rho _{nf} \left[ \frac{ \tilde{D}}{d \tilde{t}}\tilde{u}_1-\frac{ {\tilde{w}_1}^2}{\tilde{r}_1 +\tilde{R}^*} \right]= & {} - \frac{\partial \tilde{p}}{\partial \tilde{r}_1} +\mu _{nf}\Biggl (\nabla ^2 \tilde{u}_1 -\frac{\tilde{u}_1}{(\tilde{r}_1 +\tilde{R}^*)^2}-\frac{2\tilde{R}^*}{\tilde{r}_1 +\tilde{R}^*}\frac{\partial \tilde{w}_1}{\partial \tilde{z}_1}\Biggr )\nonumber \\{} & {} +\Biggl (\frac{4}{3}\frac{\partial \tilde{u}_1}{\partial \tilde{r}_1}-\frac{2}{3}\left( \frac{\tilde{R}^*}{\tilde{R}^*+\tilde{r}_1}\frac{\partial \tilde{w}_1}{\partial \tilde{z}_1}+\frac{\tilde{u}_1}{\tilde{R}^*+\tilde{r}_1}\right) \Biggr )\frac{\partial \mu _{nf}}{\partial \tilde{r}_1}. \end{aligned}$$

*Momentum* (in $z_1^*$-direction)

15 $$\begin{aligned} \rho _{nf} \left[ \frac{ \tilde{D}}{d \tilde{t}}\tilde{w}_1+ \frac{\tilde{u}_1 \tilde{w}_1}{\tilde{r}_1 +\tilde{R}^*} \right]= & {} -\left( \frac{\tilde{R}^*}{\tilde{r}_1 + \tilde{R}^*}\right) \frac{\partial \tilde{p}}{\partial \tilde{z}_1} +\mu _{nf}\Biggl (\nabla ^2 \tilde{w}_1 -\frac{\tilde{w}_1}{(\tilde{r}_1 +\tilde{R}^*)^2} +\frac{2\tilde{R}^*}{\tilde{r}_1 +\tilde{R}^*}\Biggr ) \nonumber \\{} & {} +g(\rho \beta )_{nf}(\tilde{T}-\tilde{T}_{0})+g(\rho \beta )_{nf}(\tilde{C}-\tilde{C}_{0})\nonumber \\{} & {} +\Biggl (\frac{\tilde{R}^*}{\tilde{R}^*+\tilde{r}_1}\frac{\partial \tilde{u}_1}{\partial \tilde{z}_1}+\frac{\partial \tilde{w}_1}{\partial \tilde{r}_1}-\frac{\tilde{w}_1}{\tilde{R}^*+\tilde{r}_1}\Biggr )\frac{\partial \mu _{nf}}{\partial r_1} +\rho _e E_0 -\sigma _{nf} B_0^2 \tilde{w}_1\left( \frac{\tilde{R}^*}{\tilde{r}_1 +\tilde{R}^*} \right) ^2. \end{aligned}$$

*Temperature Equation*

16 $$\begin{aligned} (\rho C_p)_{nf} \frac{ \tilde{D} \tilde{T}}{d \tilde{t}} = \kappa _{nf} \nabla ^2 \tilde{T}+ \sigma _{nf}\left( \frac{ \tilde{R}^* B_0}{\tilde{r}_1+\tilde{R}^*}\right) ^2 (\tilde{w}_1 ^2) + \sigma _{nf}E_0^2 +F_{vd} -\frac{\partial q}{\partial \tilde{r}_1}. \end{aligned}$$

*Concentration Equation*

17 $$\begin{aligned} \frac{ \tilde{D} \tilde{C}}{d \tilde{t}} = D_{m} \nabla ^2 \tilde{C}-R_b (\tilde{C}-\tilde{C}_w). \end{aligned}$$

*Electroosmotic Equation*

18 $$\begin{aligned} \nabla ^2 \tilde{\Phi } = -\frac{\tilde{\Phi }}{q^2_m} \end{aligned}$$

where $\nabla ^2 :=\frac{\partial ^2 }{\partial {\tilde{r}_1}^2} + \frac{1}{\tilde{r}_1 +\tilde{R}^*}\frac{\partial }{\partial \tilde{r}_1} + \left( \frac{\tilde{R}^*}{\tilde{r}_1 +\tilde{R}^*} \right) ^2 \frac{\partial ^2 }{\partial {\tilde{z}_1}^2}$, the material derivative is $\frac{ \tilde{D}}{d \tilde{t}}:=\frac{\partial }{\partial \tilde{t}} + \tilde{u}_1\frac{\partial }{\partial \tilde{r}_1 }+ \frac{\tilde{w}_1 \tilde{R}^* }{\tilde{r}_1 +\tilde{R}^*}\frac{\partial }{\partial \tilde{z}_1}$ and the viscous dissipation term $F_{vd}$ is given as:

19 $$\begin{aligned} F_{vd}= \mu _{nf}\biggl [2 \biggl (\frac{\partial \tilde{u}_1}{\partial \tilde{r}_1}\biggr )^2+ 2 \biggl (\frac{ \tilde{R}^* }{\tilde{r}_1+\tilde{R}^*}\frac{\partial \tilde{w}_1}{\partial \tilde{z}_1}+\frac{ \tilde{u}_1 }{\tilde{r}_1+\tilde{R}^*}\biggr )^2+ \biggl (\frac{\partial \tilde{w}_1}{\partial \tilde{r}_1} -\frac{ \tilde{w}_1 }{\tilde{r}_1+\tilde{R}^*}+\frac{ \tilde{R}^* }{\tilde{r}_1+\tilde{R}^*}\frac{\partial \tilde{u}_1}{\partial \tilde{z}_1}\biggr )^2\biggr ] \end{aligned}$$

**Table 1:** Thermophysical properties of nanofluid^65^.

Properties	Mathematical expression for nanofluid
Viscosity	$\mu _{nf}=\frac{\mu _0[1+ \gamma _{1} h(\tilde{r}_1)]}{(1-34.87(\frac{d_p}{d_f})^{-0.3} \phi _n^{1.03} )}$
Density	$\rho _{nf}=(1-\phi _n)\rho _f+\phi _n \rho _{s_1}$
Heat Capacity	$(\rho C_p)_{nf}=(1-\phi _n)(\rho C_p)_f+\phi _n (\rho C_p)_{s_1}$
Thermal Conductivity	$\frac{k_{nf}}{k_f}=\frac{k_{s_1}+(m-1) k_f-(m-1) \phi _n(k_f-k_{s_1})}{k_{s_1}+(m-1) k_f+ \phi _n(k_f-k_{s_1})}$
Electrical Conductivity	$\frac{\sigma _{nf}}{\sigma _f}=\frac{\sigma _{s_1}+(m-1) \sigma _f-(m-1) \phi _n(\sigma _f-\sigma _{s_1})}{\sigma _{s_1}+(m-1) \sigma _f+ \phi _n(\sigma _f-\sigma _{s_1})}$
Thermal Expansion Coefficient	$\gamma _{nf}=(1-\phi _n)\gamma _f+\phi _n \gamma _{s_1}$

The boundary conditions are given as:

20 $$\begin{aligned} {\left\{ \begin{array}{ll} \tilde{w}_1=0, \,\,\,\tilde{T}= \tilde{T}_0,\,\,\,\tilde{C}=\tilde{C}_0 \hspace{0.5 cm} &{}\text {at } \hspace{0.5 cm} \tilde{t}=0, \\ \tilde{w}_1=0, \,\,\, \tilde{T}=\tilde{T}_w, \,\,\, \tilde{C}=\tilde{C}_w \hspace{0.3 cm} &{}\text {at } \hspace{0.5 cm} \tilde{r}_1= \eta (\tilde{z}_1) \hspace{0.2 cm} \text {and} \hspace{0.2 cm} \tilde{r}_1= \epsilon (\tilde{z}_1). \end{array}\right. } \end{aligned}$$

The specification of boundary conditions pertaining to the potential function is as follows:

21 $$\begin{aligned} \tilde{\Phi }= & {} \tilde{\zeta }_2 \,\, \text {on} \,\, \tilde{r}_1=\epsilon (\tilde{z}_1), \nonumber \\ \tilde{\Phi }= & {} \tilde{\zeta }_1 \,\, \text {on} \,\,\tilde{r}_1=\eta (\tilde{z}_1). \end{aligned}$$

Where zeta potential functions represented by $\tilde{\zeta }_1$ and $\tilde{\zeta }_2$ are specifically denoted with respect to the arterial and catheter wall, respectively.

The pulsatile nature of blood flow is an inherent characteristic, primarily resulting from the continuous pumping action of the heart. The aforementioned phenomenon can be mathematically characterised in the subsequent manner^30,66^:

22 $$\begin{aligned} -\frac{\partial p}{\partial \tilde{z}_1} = A_0+ A_1 cos(2 \pi \omega _{p} \tilde{t}), \tilde{t}>0, \end{aligned}$$

where $A_0$ denotes the amplitude of the pressure gradient corresponding to the steady-state condition, while $A_1$ signifies the amplitude of the pressure gradient associated with the pulsatile state. The term $\tilde{\omega } = 2\pi \tilde{(\nu )}$ denotes the angular frequency pertaining to the heart.

### Non-dimensionalization

In consideration of the dimensionless parameters delineated in the nomenclature, the pertinent Eqs. (13)–(21) that govern the model under the assumption of mild stenosis $(\delta ^*<<1)$ and the condition $O(1)=\alpha = \frac{R_0}{\lambda }$ can be expressed as follows^63^:

23 $$\begin{aligned}{} & {} \frac{dp}{dr_1}=0, \end{aligned}$$

24 $$ \begin{aligned}   \frac{{\rho _{{nf}} }}{{\rho _{f} }}Re\frac{{\partial w_{1} }}{{\partial t}} &  =  - \frac{{R_{c} }}{{R_{c}  + r_{1} }}\frac{{\partial p}}{{\partial z_{1} }} + \frac{{\mu _{{nf}} }}{{\mu _{0} }}\left( {\frac{{\partial ^{2} w_{1} }}{{\partial r_{1} ^{2} }} + \frac{1}{{r_{1}  + R_{c} }}\frac{{\partial w_{1} }}{{\partial r_{1} }} - \frac{{w_{1} }}{{(r_{1}  + R_{c} )^{2} }}} \right) \\     & \quad  + U_{{hs}} q_{e}^{2} \Phi  + \frac{{(\rho \beta )_{{nf}} }}{{(\rho \beta )_{f} }}(Gr\theta _{1}  + Gc\phi _{1} ) \\     & \quad  - \frac{{m\beta _{1} h_{m} r_{1}^{{*m - 1}} }}{{\left( {1 - 34.87\left( {\frac{{d_{p} }}{{d_{f} }}} \right)^{{ - 0.3}} \phi ^{{1.03}} } \right)}}\left( {\frac{{\partial w_{1} }}{{\partial r_{1} }} - \frac{{w_{1} }}{{R_{c}  + r_{1} }}} \right) - \frac{{\sigma _{{nf}} }}{{\sigma _{f} }}\left( {\frac{{R_{c} }}{{r_{1}  + R_{c} }}} \right)^{2} M^{2} w_{1} , \\  \end{aligned}  $$

25 $$ \begin{aligned}   \frac{{(\rho C_{p} )_{{nf}} }}{{(\rho C_{p} )_{f} }}\frac{{\kappa _{f} }}{{\kappa _{{nf}} }}PrRe\frac{{\partial \theta _{1} }}{{\partial t}} &  = \frac{{\partial ^{2} \theta _{1} }}{{\partial r_{1} ^{2} }} + \frac{1}{{r_{1}  + R_{c} }}\frac{{\partial \theta _{1} }}{{\partial r_{1} }} + \frac{{\sigma _{{nf}} }}{{\sigma _{f} }}\frac{{\kappa _{f} }}{{\kappa _{{nf}} }}\left[ {\left( {\frac{{R_{c} }}{{r_{1}  + R_{c} }}} \right)^{2} BrM^{2} w_{1} ^{2}  + S_{z} } \right] + \frac{{\kappa _{f} }}{{\kappa _{{nf}} }}Nr\frac{{\partial ^{2} \theta _{1} }}{{\partial \overline{{r_{1} }} ^{2} }} \\     & \quad  + \left( {\frac{{\kappa _{f} }}{{\kappa _{{nf}} }}} \right)\left( {\frac{{\mu _{{nf}} }}{{\mu _{0} }}} \right)Br\left[ {\frac{{\partial w_{1} }}{{\partial r_{1} }} - \frac{{w_{1} }}{{r_{1}  + R_{c} }}} \right]. \\  \end{aligned}  $$

26 $$\begin{aligned}{} & {} Re Sc \frac{\partial \phi _1}{\partial t}=\frac{\partial ^2 \phi _1}{\partial r_1^2} +\frac{1}{R_c+r_1} \frac{\partial \phi _1}{\partial r_1} -Sc \xi \phi _1, \end{aligned}$$

27 $$\begin{aligned}{} & {} \frac{\partial ^2 \Phi }{\partial r_1^2} + \frac{1}{R_c+r_1}\frac{\partial \Phi }{\partial r} = q_e^2\Phi . \end{aligned}$$

Associate boundary conditions are as follows:

28 $$\begin{aligned} {\left\{ \begin{array}{ll} w_1=\theta _1=\phi _1=0 \hspace{0.5 cm} &{}\text {at } \hspace{0.5 cm} t=0,\\ w_1=0, \theta _1=1, \phi _1=1 \hspace{0.3 cm} &{}\text {at } \hspace{0.5 cm} r_1=\epsilon (z_1) \hspace{0.2 cm} \text {and} \hspace{0.2 cm} r_1=\eta (z_1). \end{array}\right. } \end{aligned}$$

Boundary condtion for electroosmotic function:

29 $$\begin{aligned} \Phi= & {} 0.1 \,\, \text {on} \,\, r_1=\epsilon (z_1),\nonumber \\ \Phi= & {} 0.3 \,\, \text {on} \,\, r_1=\eta (z_1). \end{aligned}$$

**Table 2:** Values of the physical parameters with their sources.

Parameters	Ranges	Sources
Thermal Grashof number (*Gr*)	0–6	^35,37^
Nanoparticle shape parameter (*n*)	3–8.6	^6,10^
Prandtl number (*Pr*)	0–4	^10,14^
Radiation parameter (Nr)	0–3	^37,53^
hematocrit parameter ($h_m$)	0–1	^25,34^
Magnetic Number (*M*)	0–4	^7,36^
Brinkmann number (*Br*)	0.1–2	^5,10^

The dimensionless expressions corresponding to the diseased artery are provided as: **Clot region:**

30 $$\begin{aligned} \epsilon (z_1) = {\left\{ \begin{array}{ll} c+ \sigma \exp (-\pi ^2 (z_1-z_d-1/2)^2), \,\,\,\,\,\,\,\,\,\,\,\,\,\, &{}d<z_1<d+3/2, \\ c, \,\,\,\,\,\,\,\,\,\,\,\,\,\,\,\,\,\,\,&{} \text {otherwise }, \end{array}\right. } \end{aligned}$$

**Stenosis Region:**

31 $$\begin{aligned} \eta (z_1) = {\left\{ \begin{array}{ll} 1-2\delta ^* \left[ \cos (2 \pi (\frac{z_1-d}{2}-\frac{1}{4}) -\frac{7}{100} \cos (32 \pi (z_1-d-\frac{1}{2})) \right] &{} \quad d\le z_1\le d+1, \\ 1 &{} \quad \text {otherwise }, \end{array}\right. } \end{aligned}$$

where $\sigma$ represents the maximum clot height at the axial location $z_d$, while the inner tube radius is denoted as $cR_0$, where *c* is a considerably small value $(c<< 1)$. Additionally, the maximum height of the stenosis is symbolized by parameter $\delta$ in equation (31), and the specific location of the affected segment is represented by the variable *d*.

The pressure component in dimensionless form is given as^63^:

32 $$\begin{aligned} -\frac{\partial p}{\partial z_1} = D_1(1+e \cos (c_p t)), \end{aligned}$$

where $$D_1=\frac{A_0 R_0^2}{\mu _0 U_0}, e=\frac{A_1}{A_0},$$ and $$c_p=\frac{2 \pi R_0 \omega _p}{U_0}$$.

The volumetric flow rate is defined as^10^:

33 $$\begin{aligned} Q = \int _{\epsilon }^{\eta } w_1r_1 dr_1. \end{aligned}$$

In the afflicted arterial system, the impedance encountered by the blood flow is expressed as^10^:

34 $$\begin{aligned} \lambda = \frac{L (\frac{\partial p}{\partial z_1^*})}{Q}. \end{aligned}$$

Finally, the shear stress profile is given as^49^:

35 $$\begin{aligned} \tau _{w_1}= \left( \frac{\partial w_1}{\partial r_1}\right) _{r_1=\eta }. \end{aligned}$$

### Entropy

Entropy is the measured of the irreversibility present in the system. The entropy is attribute to the change in the system cause by mass and thermal exchange. The overall entropy is the sum of entropy produces by each individual process. The dimensional volumetric entropy generation is defined as^9,67^:

36 $$\begin{aligned} E_g = \frac{\kappa _f}{\tilde{T}_0^2} \left[ \frac{\kappa _{nf}}{\kappa _f} + \frac{16 \sigma _e \tilde{T}_0{^3}}{3 k_e \kappa _f}\right] \left( \frac{\partial \tilde{T}}{\partial r_1}\right) ^2 + \frac{\mu _{nf}}{\tilde{T}_0} \left( \frac{\partial \tilde{w}_1}{\partial r_1}\right) ^2 + \frac{\sigma _{nf}}{\tilde{T}_0} \left( \left( \frac{\tilde{R}^*}{\tilde{r}_1 +\tilde{R}^*} \right) B_0 \tilde{w}_1-E_0 \right) ^2 +\frac{D_b}{\tilde{C}_w}\left( \frac{\partial \tilde{C}}{\partial r_1}\right) ^2. \end{aligned}$$

There are four components in the above equations. The first term on right hand side depicts the irreversibility due to heat transfer , the second term for the hydromagnetic, third term for the fluid friction and the last term for solute irreversibility. We simplified the above equation further, to get;

37 $$\begin{aligned} E_g= & {} \frac{\kappa _f}{\tilde{T}_0^2}\frac{(\tilde{T}_w-\tilde{T}_0)^2}{R_0^2} \biggl \{ \left[ \frac{\kappa _{nf}}{\kappa _f} + \frac{16 \sigma _e \tilde{T}_0^3}{3 k_e \kappa _f}\right] \left( \frac{\partial \theta _1}{\partial r_1}\right) ^2 + \frac{\mu _{nf} U_0^2 }{\kappa _f \Delta \tilde{T}} \left( \frac{\partial w_1}{\partial r_1}\right) ^2\nonumber \\{} & {} + \frac{\sigma _{nf} B_0^2 U_0 \tilde{T}_0 R_0^2 }{\kappa _f \Delta \tilde{T}} \left( \left( \frac{R_c}{r_1+R_c}\right) w_1- \frac{E_0}{B_0 U_0}\right) ^2 \nonumber \\{} & {} +\frac{D_b}{\tilde{C}_w} \left( \frac{\Delta \tilde{C}}{R_0}\right) ^2 \frac{\tilde{T}_0^2 }{\kappa _f}\frac{R_0^2}{\Delta \tilde{T} } \left( \frac{\partial \phi _1}{\partial r_1}\right) ^2 \biggr \}. \end{aligned}$$

The dimensionless $N_s$ defined as the ratio of total entropy generation to characteristic entropy transfer. It is defined as $N_s=\frac{\tilde{T}_0^2 R_0^2}{\kappa _f (\Delta \tilde{T})^2} \times E_g$. Using equation above, we have

38 $$\begin{aligned} N_s = \left[ \frac{\kappa _{nf}}{\kappa _f} + Nr\right] \left( \frac{\partial \theta _1}{\partial r_1}\right) ^2 + \frac{\mu _{nf}}{\mu _0} \Bigg \{ \left( \frac{\partial w_1}{\partial r_1}\right) ^2\Bigg \}\frac{Br}{\Omega } +\frac{\sigma _{nf}}{\sigma _{f}} \frac{M^2 Br}{\Omega } \left( \left( \frac{R_c}{r_1+R_c}\right) w_1-E_1 \right) ^2 + \frac{\Lambda \Gamma }{\Omega } \left( \frac{\partial \phi _1}{\partial r_1}\right) ^2, \end{aligned}$$

where $\Gamma = \frac{D_b \tilde{T}_0 \Delta \tilde{C}}{ \kappa _f \Delta \tilde{T} }$. The Bejan number is defined as the ratio of heat transfer irreversibility to total irreversibility. So, we have

39 $$\begin{aligned} Be= & {} \frac{N}{N_s}= \frac{\left[ \frac{\kappa _{nf}}{\kappa _f} + Nr\right] (\frac{\partial \theta _1}{\partial r})^2}{\left[ \frac{\kappa _{nf}}{\kappa _f} + Nr\right] \left( \frac{\partial \theta _1}{\partial r_1}\right) ^2 + \frac{\mu _{nf}}{\mu _0} \Bigg \{ \left( \frac{\partial w_1}{\partial r_1}\right) ^2\Bigg \}\frac{Br}{\Omega } +\frac{\sigma _{nf}}{\sigma _{f}} \frac{M^2 Br}{\Omega } \left( \left( \frac{R_c}{r_1+R_c}\right) w_1-E_1 \right) ^2 + \frac{\Lambda \Gamma }{\Omega } \left( \frac{\partial \phi _1}{\partial r_1}\right) ^2}. \end{aligned}$$

## Numerical methodology

The mathematical model under consideration yields a set of non-linear coupled PDEs (partial differential equations), for which obtaining exact solutions proves to be challenging. In all but a few very basic circumstances, accurate solutions to these equations are impossible. As a result, several different numerical techniques have been developed to address these problems.

**Figure 2:**
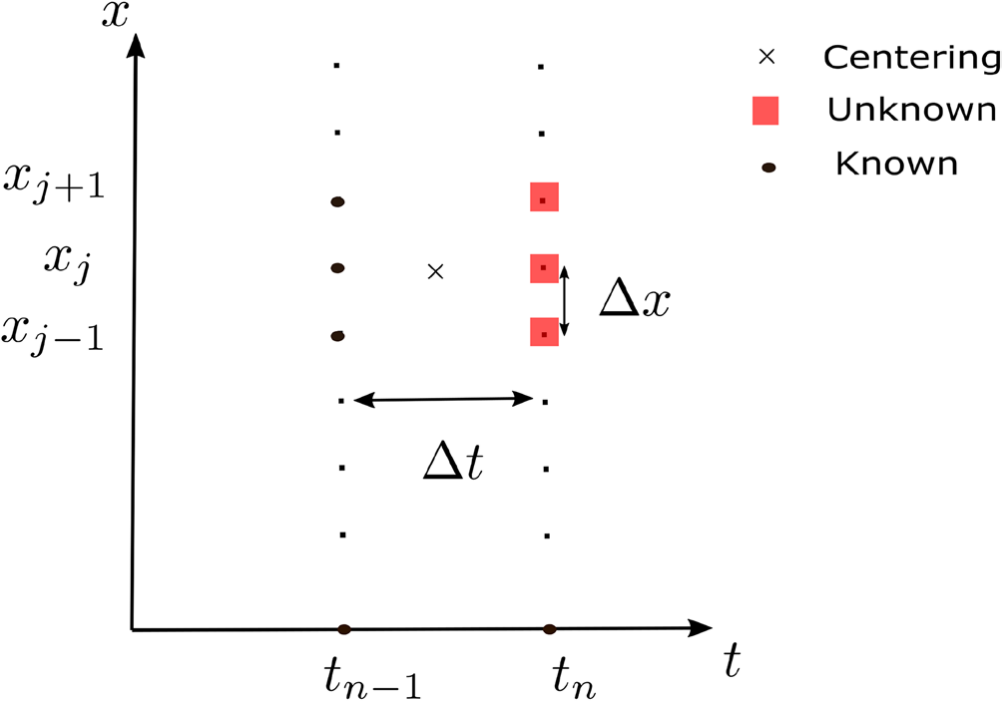
Grid for the Crank–Nicolson scheme.

With today’s fast computers and cutting-edge commercial software, such methods have become more straightforward and accurate. There have been a number of researchers that have suggested the Crank–Nicolson technique as an implicit strategy. It is second-order convergent in time. This method uses the finite difference grid as shown in Fig. 2 , replacing the spatial derivative at $\left( t_{n-{1}/{2}},x_j \right)$ by taking the average of upstream and downstream values at $t_{n-1}$ and $t_{n}$, respectively. In a similar manner, the time derivative can be substituted with the central difference formula at the point $\left( t_{n-{1}/{2}},x_j \right)$.

### Discretization of governing equations

**Figure 3:**
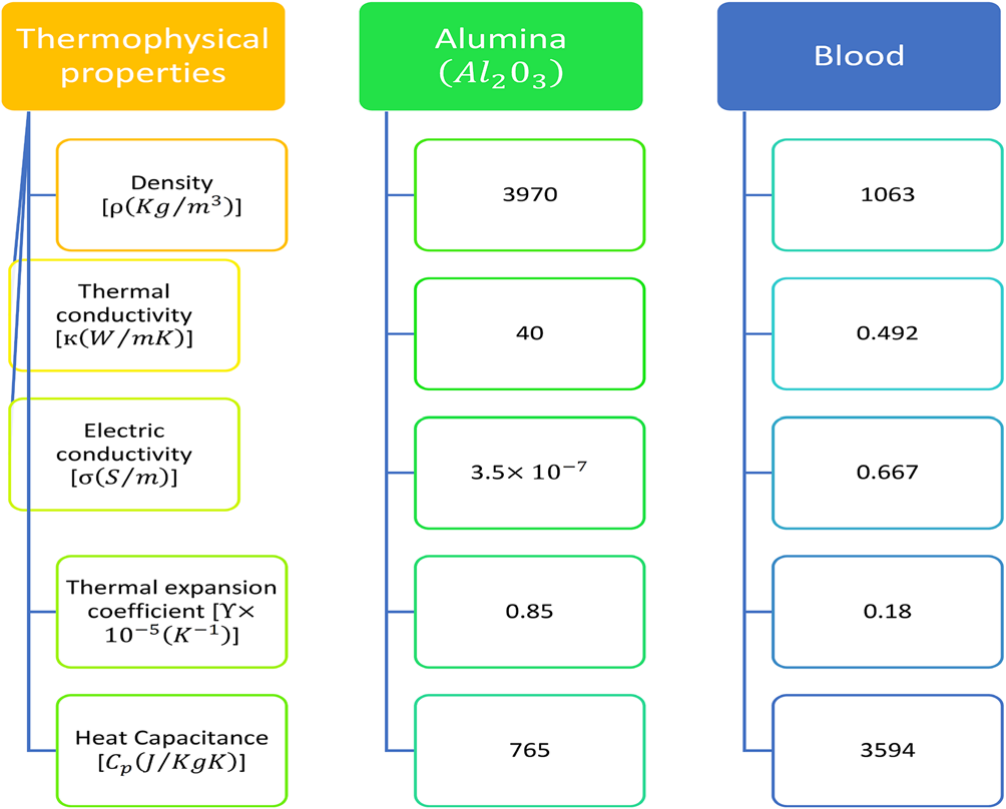
Thermophysical properties of blood and nanoparticle.

In this study, we utilized the dimensional parameter and thermophysical parameters specified in the nomenclature and Table 1, respectively. Additionally, the thermophysical properties of blood and nanoparticles, as presented in Figure 3, were taken into consideration, along with the physical parameters from Table 2. The resulting discretized governing equations are displayed as follows:

40 $$\begin{aligned}{} & {} \bigg [(1-\phi _n)+\phi _n \frac{\rho _{s_1}}{\rho _{f}}\bigg ]Re \bigg [\frac{\bar{w}_i^{k+1}-\bar{w}_i^{k}}{\Delta t}\bigg ] =\frac{R_c}{R_c+r(i)}D_1[1+e \cos (c_p t^k)]\nonumber \\{} & {} \quad + \frac{[1+\beta _1h_m(1-(\frac{r(i)}{R_0})^m)]}{(1-34.87 \left( \frac{d_p}{d_f}\right) ^{-0.3}\phi _{n}^{1.03})} \bigg [\frac{1}{2}\bigg (\frac{\bar{w}_{i+1}^{k+1}-2\bar{w}_i^{k+1}+\bar{w}_{i-1}^{k+1}}{(\Delta x)^2}+\frac{\bar{w}_{i+1}^{k}-2\bar{w}_i^{k}+\bar{w}_{i-1}^{k}}{(\Delta x)^2}\bigg )\nonumber \\{} & {} \quad +\frac{1}{4(R_c+r(i))}\bigg (\frac{ \bar{w}_{i+1}^{k+1}-\bar{w}_{i-1}^{k+1}}{\Delta x}\nonumber \\{} & {} \quad +\frac{\bar{w}_{i+1}^{k}-\bar{w}_{i-1}^{k}}{\Delta x}\bigg )-\frac{(\bar{w}_i^k+\bar{w}_i^{k+1})}{2(R_c+r(i))^2}\bigg ]-\frac{m\beta _1h_m (r(i))^{m-1}}{(1-34.87 \left( \frac{d_p}{d_f}\right) ^{-0.3}\phi _{n}^{1.03})}\nonumber \\{} & {} \quad \bigg [\frac{(\bar{w}_{i+1}^{k+1}-\bar{w}_{i-1}^{k+1}+\bar{w}_{i+1}^{k}-\bar{w}_{i-1}^{k})}{4\Delta x} -\frac{(\bar{w}_i^k+\bar{w}_i^{k+1})}{2(R_c+r(i))} \bigg ]+\nonumber \\{} & {} \quad \bigg [(1-\phi _{n})+\phi _{n} \frac{(\rho \beta )_{s1}}{(\rho \beta )_{f}}\bigg ](Gr \theta _i^k +Gc \phi _i^k) -\frac{1}{2}\frac{\sigma _{hnf}}{\sigma _{f}} M^2 (\bar{w}_i^k+\bar{w}_i^{k+1}) \left( \frac{R_c}{R_c+r(i)}\right) ^2 +U_{hs}q^2 \Phi _i^k, \end{aligned}$$

41 $$\begin{aligned}{} & {} \bigg [(1-\phi _n)+\phi _n \frac{(\rho C_p)_{s1}}{(\rho C_p)_{f}}\bigg ] \bigg [\frac{\theta _i^{k+1}-\theta _i^{k}}{dt}\bigg ] =\frac{1}{RePr}\frac{\kappa _{hnf}}{\kappa _f}\bigg [\bigg (\frac{\theta _{i+1}^{k+1}-2\theta _i^{k+1}+\theta _{i-1}^{k+1}}{2 (\Delta x)^2} +\frac{\theta _{i+1}^{k}-2\theta _i^{k}+\theta _{i-1}^{k}}{2 (\Delta x)^2}\bigg )\nonumber \\{} & {} \quad +\frac{1}{4(R_c+r(i))}\bigg (\frac{ \theta _{i+1}^{k+1}-\theta _{i-1}^{k+1}}{\Delta x}+\frac{\theta _{i+1}^{k}-\theta _{i-1}^{k}}{\Delta x}\bigg )\bigg ]\nonumber \\{} & {} \quad +\frac{Nr}{RePr}\bigg [\frac{1}{2}\bigg (\frac{\theta _{i+1}^{k+1}-2\theta _i^{k+1}+\theta _{i-1}^{k+1}}{(\Delta x)^2} +\frac{\theta _{i+1}^{k}-2\theta _i^{k}+\theta _{i-1}^{k}}{(\Delta x)^2}\bigg )\bigg ]\nonumber \\{} & {} \quad +\biggl (\frac{\mu _{nf}}{\mu _0}\biggr ) \biggl (\frac{Br}{Re Pr}\biggr )\left[ \frac{\bar{w}_{i+1}^{k+1}-\bar{w}_{i-1}^{k+1}+\bar{w}_{i+1}^{k}-\bar{w}_{i-1}^{k}}{4 \Delta x}-\frac{\bar{w}_{i}^{k}+\bar{w}_{i}^{k+1}}{2(R_c+r(i))}\right] ^2\nonumber \\{} & {} \quad +\frac{1}{Pr Re}\left\{ \frac{\sigma _{nf}}{\sigma _f} \frac{\kappa _f}{\kappa _{nf}} \left[ \left( \frac{R_c}{R_c+r(i)}\right) ^2BrM^2(\bar{w}_{i}^2)^{k} +S_z\right] \right\} , \end{aligned}$$

42 $$\begin{aligned}{} & {} \left[ \frac{\phi _{i}^{k+1}-\phi _{i}^{k}}{\Delta t}\right] = \frac{1}{ReSc} \bigg [\bigg (\frac{\theta _{i+1}^{k+1}-2\theta _i^{k+1}+\theta _{i-1}^{k+1}}{2 (\Delta x)^2}\nonumber \\{} & {} \quad +\frac{\theta _{i+1}^{k}-2\theta _i^{k}+\theta _{i-1}^{k}}{2 (\Delta x)^2}\bigg ) +\frac{1}{4(R_c+r(i))}\bigg (\frac{ \theta _{i+1}^{k+1}-\theta _{i-1}^{k+1}}{\Delta x}+\frac{\theta _{i+1}^{k}-\theta _{i-1}^{k}}{\Delta x}\bigg )\bigg ]\nonumber \\{} & {} \quad -\frac{\xi }{2Re} (\phi _i^k+\phi _i^{k+1}) \end{aligned}$$

43 $$\begin{aligned}{} & {} \frac{\Phi _{i+1}-2\Phi _i+\Phi _{i-1}}{h^2} + \frac{1}{R_c +r(i)} \{\frac{\Phi _{i+1}-\Phi _{i-1}}{2h} \} =q^2 \Phi _{i}. \end{aligned}$$

Here, $\phi _n$ denotes the nanoparticles volumetric concentration. The discretized equations for the initial and boundary conditions are given as:

44 $$\begin{aligned} \bar{w}_{1}^{k+1}&=0, \theta _{1}^{k+1}=1, \phi _{1}^{k+1}=1, \quad \bar{w}_{N+1}^{k+1}=0,\theta _{N+1}^{k+1}=1, \phi _{N+1}^{k+1}=1, \nonumber \\ \bar{w}_{i}^{1}&=0, \theta _{i}^{1}=0, \phi _{i}^{1}=0, \quad \Phi _1=0.1, \Phi _{N+1}=0.3. \end{aligned}$$

The tri-diagonal system obtained from Eq. (40) is written as:

45 $$\begin{aligned} R_i^k \bar{w}_{i-1}^{k+1} + S_i^k \bar{w}_{i}^{k+1} + T_{i}^k \bar{w}_{i+1}^{k+1} = R_i^{'k} \bar{w}_{i-1}^{k} + S_i^{'k} \bar{w}_i^{k} + T_i^{'k} \bar{w}_{i+1}^{k}+F_i^k, \end{aligned}$$

where

46 $$ \begin{aligned}   T_{i}^{k}  &  =  - \frac{{\left[ {1 + \beta _{1} h_{m} \left( {1 - \left( {\frac{r}{{R_{0} }}} \right)^{m} } \right)} \right]}}{{(1 - 34.87\left( {\frac{{d_{p} }}{{d_{f} }}} \right)^{{ - 0.3}} \phi _{n}^{{1.03}} )}}\left( {\frac{{\Delta t}}{{2(\Delta x)^{2} }}\quad  + \frac{{\Delta t}}{{4(R_{c}  + r(i))}}} \right) \\     & \quad  + \frac{{m\beta _{1} h_{m} (r(i))^{{m - 1}} }}{{\left( {1 - 34.87\left( {\frac{{d_{p} }}{{d_{f} }}} \right)^{{ - 0.3}} \phi _{n}^{{1.03}} } \right)}}\left( {\frac{{\Delta t}}{{4\Delta x}}} \right), \\  \end{aligned}  $$

47 $$ \begin{aligned}   S_{i}^{k}  &  = Re\left[ {(1 - \phi _{n} ) + \phi _{n} \frac{{\rho _{{s1}} }}{{\rho _{f} }}} \right] - \frac{{\left[ {1 + \beta _{1} h_{m} (1 - (\frac{r}{{R_{0} }})^{m} )} \right]}}{{\left( {1 - 34.87\left( {\frac{{d_{p} }}{{d_{f} }}} \right)^{{ - 0.3}} \phi _{n}^{{1.03}} } \right)}}\left( { - \frac{{\Delta t}}{{(\Delta x)^{2} }} - \frac{{\Delta t}}{{2(R_{c}  + r(i))^{2} }}} \right) \\     & \quad  - \frac{{m\beta _{1} h_{m} (r(i))^{{m - 1}} }}{{\left( {1 - 34.87\left( {\frac{{d_{p} }}{{d_{f} }}} \right)^{{ - 0.3}} \phi _{n}^{{1.03}} } \right)}}\left( {\frac{{dt}}{{2(R_{c}  + r(i))}}} \right) + \frac{{\Delta t}}{2}\frac{{\sigma _{{nf}} }}{{\sigma _{f} }}M^{2} \left( {\frac{{R_{c} }}{{R_{c}  + r(i)}}} \right)^{2} , \\  \end{aligned}  $$

48 $$\begin{aligned}{} & {} R_i^k=-\frac{[1+\beta _1h_m(1-(\frac{r}{R_0})^m)]}{(1-34.87 \left( \frac{d_p}{d_f}\right) ^{-0.3}\phi _{n}^{1.03})}\bigg (\frac{\Delta t}{2 (\Delta x)^2}-\frac{\Delta t}{4(R_c+r(i))}\bigg )\nonumber \\{} & {} \quad +\frac{m\beta _1h_m (r(i))^{m-1}}{(1-34.87 \left( \frac{d_p}{d_f}\right) ^{-0.3}\phi _{n}^{1.03})}\bigg (-\frac{\Delta t}{4 \Delta x}\bigg ), \end{aligned}$$

49 $$ \begin{aligned}   T_{i}^{{\prime k}}  &  = \frac{{\left[ {1 + \beta _{1} h_{m} \left( {1 - \left( {\frac{r}{{R_{0} }}} \right)^{m} } \right)} \right]}}{{\left( {1 - 34.87\left( {\frac{{d_{p} }}{{d_{f} }}} \right)^{{ - 0.3}} \phi _{n}^{{1.03}} } \right)}}\left( {\frac{{\Delta t}}{{2(\Delta x)^{2} }} + \frac{{\Delta t}}{{4(R_{c}  + r(i))}}} \right) \\     & \quad  - \frac{{m\beta _{1} h_{m} (r(i))^{{m - 1}} }}{{\left( {1 - 34.87\left( {\frac{{d_{p} }}{{d_{f} }}} \right)^{{ - 0.3}} \phi _{n}^{{1.03}} } \right)}}\left( {\frac{{\Delta t}}{{4\Delta x}}} \right), \\  \end{aligned}  $$

50 $$ \begin{aligned}   S_{i}^{{\prime k}}  &  = Re\left[ {(1 - \phi _{n} ) + \phi _{n} \frac{{\rho _{{s1}} }}{{\rho _{f} }}} \right] + \frac{{[1 + \beta _{1} h_{m} (1 - (\frac{r}{{R_{0} }})^{m} )]}}{{\left( {1 - 34.87\left( {\frac{{d_{p} }}{{d_{f} }}} \right)^{{ - 0.3}} \phi _{n}^{{1.03}} } \right)}}\left( { - \frac{{\Delta t}}{{(\Delta x)^{2} }} - \frac{{\Delta t}}{{2(R_{c}  + r(i))^{2} }}} \right) \\     & \quad  + \frac{{m\beta _{1} h_{m} (r(i))^{{m - 1}} }}{{\left( {1 - 34.87\left( {\frac{{d_{p} }}{{d_{f} }}} \right)^{{ - 0.3}} \phi _{n}^{{1.03}} } \right)}}\left( {\frac{{\Delta t}}{{2(R_{c}  + r(i))}}} \right), \\  \end{aligned}  $$

51 $$ \begin{aligned}   R_{i}^{{\prime k}}  &  = \frac{{\left[ {1 + \beta _{1} h_{m} \left( {1 - \left( {\frac{r}{{R_{0} }}} \right)^{m} } \right)} \right]}}{{\left( {1 - 34.87\left( {\frac{{d_{p} }}{{d_{f} }}} \right)^{{ - 0.3}} \phi _{n}^{{1.03}} } \right)}}\left( {\frac{{\Delta t}}{{2(\Delta x)^{2} }} - \frac{{\Delta t}}{{4(R_{c}  + r(i))}}} \right) \\     & \quad  + \frac{{m\beta _{1} h_{m} (r(i))^{{m - 1}} }}{{\left( {1 - 34.87\left( {\frac{{d_{p} }}{{d_{f} }}} \right)^{{ - 0.3}} \phi _{n}^{{1.03}} } \right)}}\left( {\frac{{\Delta t}}{{4\Delta x}}} \right), \\  \end{aligned}  $$

52 $$\begin{aligned}{} & {} F_i^k=\Delta t \frac{D_1R_c}{(R_c+r(i))}[1+e \cos (c_1 t^k)]+(\Delta t) \bigg [(1-\phi _{n})+\phi _{n} \frac{(\rho \beta )_{s_1}}{(\rho \beta )_{f}}\bigg ] (Gr \theta _i^k+Gc \phi _i^k)+\Delta t U_{hs}q^2 \Phi _i^k. \end{aligned}$$

Using Eq. (41), we may derive the tridiagonal system:

53 $$\begin{aligned} A_i^k\theta _{i-1}^{k+1}+B_i^k\theta _i^{k+1}+C_i^k\theta _{i+1}^{k+1}=A_i^{'k}\theta _{i-1}^{k}+B_i^{'k}\theta _i^{k}+C_i^{'k}\theta _{i+1}^{k}+D_i^k, \end{aligned}$$

where

54 $$\begin{aligned} A_i^k= & {} -\frac{1}{Re Pr}\frac{\kappa _{nf}}{\kappa _f}\bigg (\frac{\Delta t}{2 (\Delta x)^2}-\frac{1}{4(R_c+r(i))}\frac{\Delta t}{\Delta x}\bigg )-\frac{Nr}{2Re Pr}\bigg (\frac{\Delta t}{(\Delta x)^2}\bigg ), \end{aligned}$$

55 $$\begin{aligned} B_i^k= & {} \bigg [(1-\phi _n)+\phi _n \frac{(\rho C_p)_{s_1}}{(\rho C_p)_{f}}\bigg ]+\frac{1}{Re Pr}\frac{\kappa _{nf}}{\kappa _f}\frac{\Delta t}{(\Delta x)^2}+\frac{Nr}{Re Pr}\bigg (\frac{\Delta t}{(\Delta x)^2}\bigg ), \end{aligned}$$

56 $$\begin{aligned} C_i^k= & {} -\frac{1}{Re Pr}\frac{\kappa _{nf}}{\kappa _f}\bigg (\frac{\Delta t}{2 (\Delta x)^2}+\frac{1}{4(R_c+r(i))}\frac{\Delta t}{(\Delta x)}\bigg )-\frac{Nr}{2Re Pr}\bigg (\frac{\Delta t}{(\Delta x)^2}\bigg ), \end{aligned}$$

57 $$\begin{aligned} A_i^{'k}= & {} \frac{1}{Re Pr}\frac{\kappa _{nf}}{\kappa _f}\bigg (\frac{\Delta t}{2 (\Delta x)^2}-\frac{1}{4(R_c+r(i))}\frac{\Delta t}{\Delta x}\bigg )+\frac{Nr}{2Re Pr}\bigg (\frac{\Delta t}{(\Delta x)^2}\bigg ), \end{aligned}$$

58 $$\begin{aligned} B_i^{'k}= & {} \bigg [(1-\phi _n)+\phi _n \frac{(\rho C_p)_{s1}}{(\rho C_p)_{f}}\bigg ]-\frac{1}{Re Pr}\frac{\kappa _{nf}}{\kappa _f}\frac{\Delta t}{(\Delta x)^2}-\frac{Nr}{Re Pr}\bigg (\frac{\Delta t}{(\Delta x)^2}\bigg ), \end{aligned}$$

59 $$\begin{aligned} C_i^{'k}= & {} \frac{1}{Re Pr}\frac{\kappa _{nf}}{\kappa _f}\bigg (\frac{\Delta t}{2 (\Delta x)^2}+\frac{1}{4(R_c+r(i))}\frac{\Delta t}{\Delta x}\bigg )+\frac{Nr}{2Re Pr}\bigg (\frac{\Delta t}{(\Delta x)^2}\bigg ) \end{aligned}$$

60 $$\begin{aligned} D_i^k= & {} \biggl (\frac{\mu _{nf}}{\mu _0}\biggr ) \biggl (\frac{Br \Delta t}{Re Pr}\biggr )\biggl [\frac{w_{i+1}^{k+1}-w_{i-1}^{k+1}+w_{i+1}^{k}-w_{i-1}^{k}}{4 \Delta x}-\frac{w_{i}^{k}+w_{i}^{k+1}}{2(R_c + r(i))}\biggr ]^2\nonumber \\{} & {} + \frac{\Delta t}{Re Pr} \left[ \frac{\sigma _{nf}}{\sigma _f}\left( \frac{Rc}{Rc+r(i)}\right) ^2 Br M^2 (w_i^{k})^2+S_{z}\right] . \end{aligned}$$

Using Eq. (42), we may derive the tridiagonal system as:

61 $$\begin{aligned} X_i^k \phi _{i-1}^{k+1}+Y_i^k \phi _i^{k+1}+Z_i^k \phi _{i+1}^{k+1}=X_i^{'k}\phi _{i-1}^{k}+Y_i^{'k}\phi _i^{k}+Z_i^{'k}\phi _{i+1}^{k}+E_i^k, \end{aligned}$$

where

62 $$\begin{aligned} X_i^k= & {} \frac{1}{ReSc}\left[ -\frac{\Delta t}{2 (\Delta x)^2}+\frac{\Delta t }{4 \Delta x}\frac{1}{(Rc+r(i))}\right] , \end{aligned}$$

63 $$\begin{aligned} Y_i^k= & {} \frac{1}{ReSc}\left[ ReSc+\frac{\Delta t}{2 (\Delta x)^2}+Sc\xi \frac{\Delta t }{2}\right] , \end{aligned}$$

64 $$\begin{aligned} Z_i^k= & {} \frac{1}{ReSc}\left[ -\frac{\Delta t}{2 (\Delta x)^2}-ReSc\frac{\Delta t }{4 \Delta x}\frac{1}{(Rc+r(i))}\right] , \end{aligned}$$

65 $$\begin{aligned} X_i^{'k}= & {} \frac{1}{ReSc}\left[ \frac{\Delta t}{2 (\Delta x)^2}-\frac{\Delta t }{4 \Delta x}\frac{1}{(Rc+r(i))}\right] , \end{aligned}$$

66 $$\begin{aligned} Y_i^{'k}= & {} \frac{1}{ReSc}\left[ ReSc-\frac{\Delta t}{2h^2}-Sc\xi \frac{\Delta t }{2}\right] , \end{aligned}$$

67 $$\begin{aligned} Z_i^{'k}= & {} \frac{1}{ReSc}\left[ \frac{\Delta t}{2 (\Delta x)^2}+ReSc\frac{\Delta t }{4 \Delta x}\frac{1}{(Rc+r(i))}\right] ,\,\,\,E_i^k=0. \end{aligned}$$

The flow region has been partitioned into a grid composed of $(N+1) \times (M+1)$ points. In our analysis, we have chosen to utilise temporal and spatial discretization with step sizes $\Delta t=0.01$ and $\Delta x = 0.001$, respectively, while considering the Crank–Nicolson method, which is renowned for its second-order convergence.

In order to enhance the precision of our results, we have implemented a meshing scheme that ensures the attainment of a convergent solution. In order to perform numerical computations, a custom MATLAB code has been developed to solve for the distribution of velocity, temperature, electroosmotic, and concentration fields within the specified domain. It is worth noting that the electroosmotic equation is unaffected by changes in time, enabling us to effectively create a specialised function file that includes it in each temporal iteration.

## Result and discussion

### Validation

**Figure 4:**
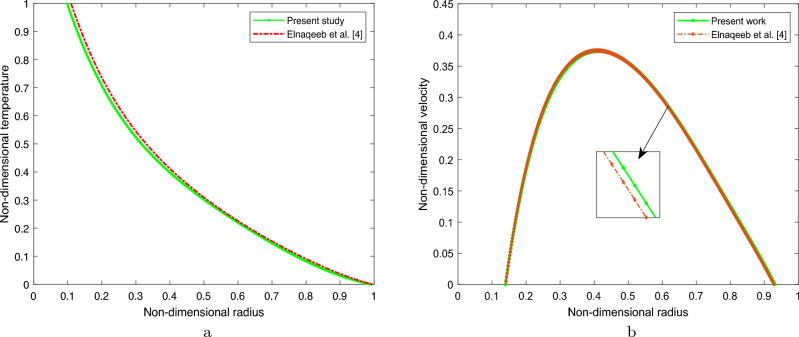
Comparision for (**a**) Temperature distribution for $\sigma =0.2$ , (**b**) Velocity distribution for $Gr=5$.

This study aims to validate our model by comparing it to the previously published research conducted by Elnaqeeb et al.^4^. For the validation process, the radiation conditions and irregular stenosis were not considered. To validate the findings, the primary objective was to analyse the behaviour of copper nanoparticles within the bloodstream when subjected to a straight artery ($R_c \ne 0$) while accounting for the source term in place of radiation. The boundary conditions utilised in this study for validation process were derived from the previously mentioned research conducted by Elnaqeeb et al.^4^. The temperature and velocity profiles for the fixed parameters $\sigma =0.2$ and $Gr=5$ are illustrated in Fig. 4a,b, correspondingly. Significantly, the findings derived from this investigation demonstrate a substantial degree of concurrence with the outcomes presented in the research conducted by Elnaqeeb et al. ^4^.

### Velocity profile

**Figure 5:**
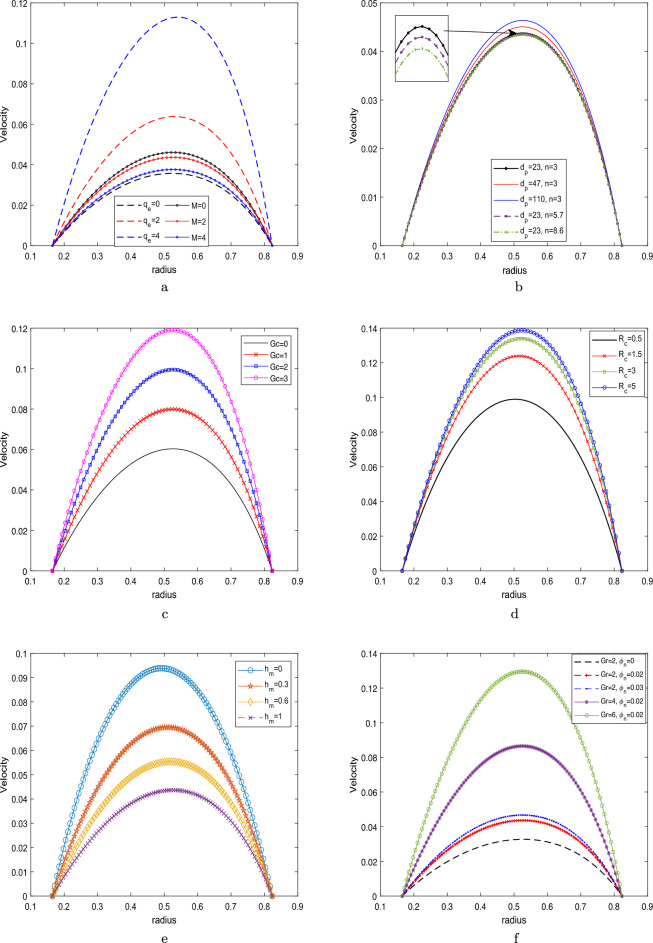
Velocity profile by varying (**a**) *M* and $q_e$,     (**b**) $d_p$ and *n*,     (**c**) *Gc*,     (**d**) $R_c$,     (**e**) $h_m$,     (**f**) *Gr* and nanoparticle concentration $\phi _n$.

Figure 5 depicts the impact of the different parameters on the velocity profile. The impact of magnetic field parameters and electric kinetic potential between the clot and stenotic zone is depicted in Fig. 5a. The analysis reveals that an increase in the magnetic field parameter leads to a decrease in the velocity profile, whereas a contrasting trend is observed in relation to the parameter $q_e$. The Debye–Huckel parameter $q_e$ exerts a substantial influence on the fluid motion throughout the entire region being analysed. The figure provided depicts the scenarios when an electric double layer (EDL) is absent ($q_e = 0$) and when it is present ($q_e \ne 0$). It is worth mentioning that an augmentation in the electro-kinetic parameter, which corresponds to a decrease in the electrical double layer (EDL) thickness, enhances the movement of fluid by reducing the drag force acting on it. The bulk fluid moves proportionately to the charged surface due to the applied electric field. Increasing the magnetic field parameter from $M=0$ to $M=4$ resists the fluid motion due to resistive Lorentz force. Figure 5b elucidates the effect of the shape and size of the nanoparticles on the velocity profile. According to several researchers, the velocity profile increases as particle size grows from 23 nm to 110 nm. The surface area ratio of a nanoparticle increases with nanoparticle size. Thus, reducing the nanoparticle size enhances the fluid’s viscosity and impedes the fluid flow. Figure 5c depicts the increasing trend of the velocity profile for an enhancing *Gc* parameter $(Gc=0,1,2,3)$. The concentration profile is coupled with the velocity profile as seen in Eq. (24).

The solutal Grashof paramter is the ratio of solutal buoyancy with the hemodynamic viscous force. The buoyancy force depicts the dominant behavior as the value of *Gc* parameter increases and thus, shows the increasing the velocity profile. Figure 5d demonstrates the effect of curvature on the velocity profile. It is observed from the graph that the velocity enhances as the curvature parameter increases from 0 to 5. This phenomenon indicates that as the curvature parameter increases, the artery tends to transform into a straight channel, resulting in reduced fluid obstruction near the wall and facilitating fluid motion. Figure 5e illustrates the velocity profile by varying the hematocrit dependent viscosity parameter. In the current study, the figure illustrates both scenarios, one with negligible viscosity ($h_m=0$) and the other with varying viscosity ($h_m \ne 0$). The velocity profile demonstrates a decrease as the hematocrit parameter increases, primarily due to the concurrent increase in fluid viscosity. The velocity profile, as depicted in Fig. 5f, showcases the cumulative effect of nanoparticle volumetric concentration and the Grashof number (*Gr*). It presents a comparative analysis between the velocity profiles of pure blood (devoid of any added nanoparticles) and $\hbox {Al}_2\hbox {O}_3$-blood (containing integrated nanoparticles). One can observed from the figure that the velocity distribution improves as the Grashof number or nanoparticles concentration enhances in the blood. Enhancing the Grashof number increases the velocity profile due to the dominating buoyancy force over the viscous force.

### Temperature profile and concentration profile

**Figure 6:**
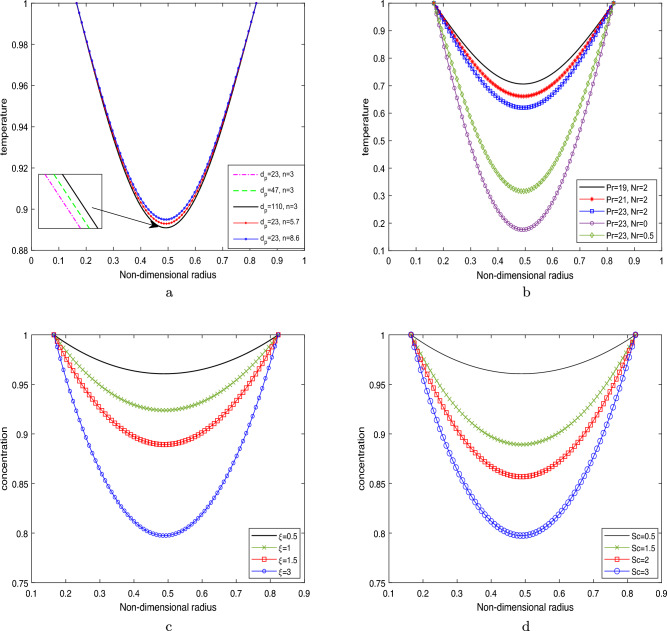
Temperature profile by varying (**a**) $d_p$ and *n*,    (**b**) *Pr* and *Nr*;     Variation in concentration profile by varying (**c**) $\xi$,     (**d**) *Sc*.

The temperature profile enhancement is depicted in Fig. 6a, illustrating its dependence on the nanoparticle size $d_p$ and the shape parameter. The shape parameter is denoted by *n*, where $n=3$ corresponds to spheres, $n=5.7$ and $n=8.6$ represents for platelets and bricks, respectively.

The findings suggest that the shape parameter significantly affects the temperature profile, while the size parameter has a relatively minimal impact. It is worth noting that an augmentation in the shape parameter enhances thermal conductivity, thereby resulting in an elevated temperature profile. The investigation focuses on the influence of two key parameters, namely the Prandtl number (*Pr*) and the radiation parameter (*Nr*), on the temperature profile within the context of Fig. 6b. The provided figures demonstrate a noticeable increase in the temperature distribution as the radiation parameter (*Nr*) progresses from $Nr=0$ (representing the absence of radiation) to $Nr=2$. The observed escalation in temperature distribution can be ascribed to an accompanying surge in the generation of thermal energy, thereby contributing to an upward trajectory in the temperature profile. Therefore, *Nr* is considered to be a critical factor in determining the temperature profile. The implications of the findings presented in this study are of considerable importance across multiple domains. These endeavours encompass the observation of temperature elevations during hyperthermia therapy for cancer and the advancement of drug administration mechanisms that employ magnetically altered nanoparticles for damaged arterial structures. In certain pathological scenarios, surgeons may opt to administer a heightened dosage of radiation to enhance the thermal distribution, thereby selectively focusing on malignant cells while safeguarding the integrity of healthy ones. Moreover, the analysis presented in Fig. 6b demonstrates that an augmentation in the Prandtl number (*Pr*) from 19 to 25 leads to a more advantageous thermal profile. The observed occurrence can be ascribed to the inverse correlation between the Prandtl number and the effective thermal conductivity. It may be noted that the rate at which heat is transmitted from the artery walls to the surrounding fluid (blood) is reduced for higher Prandtl numbers. This particular observation has the potential to play a crucial role in enhancing the efficiency of heat transfer mechanisms within a range of biomedical contexts.

The fact that the concentration profile has a suppressing effect for an increasing value in the chemical reaction parameter may be gleaned from Fig. 6c, as shown. This has occurred as a consequence of the low molecular diffusivity that rises as the value of $\xi$ increases; as a result, less fluid diffuses through the artery wall. Therefore, this behaviour manifests itself everywhere across the flow field. The effect of Schmidt number on the concentration profile is illustrated in Fig. 6d. It is possible to deduce from the figure that the concentration profile will get lower as the Schmidt number gets higher. As the Schmidt number increases, there will be less mass diffusion, resulting in a lower concentration profile.

### Flow rate and impedance

**Figure 7:**
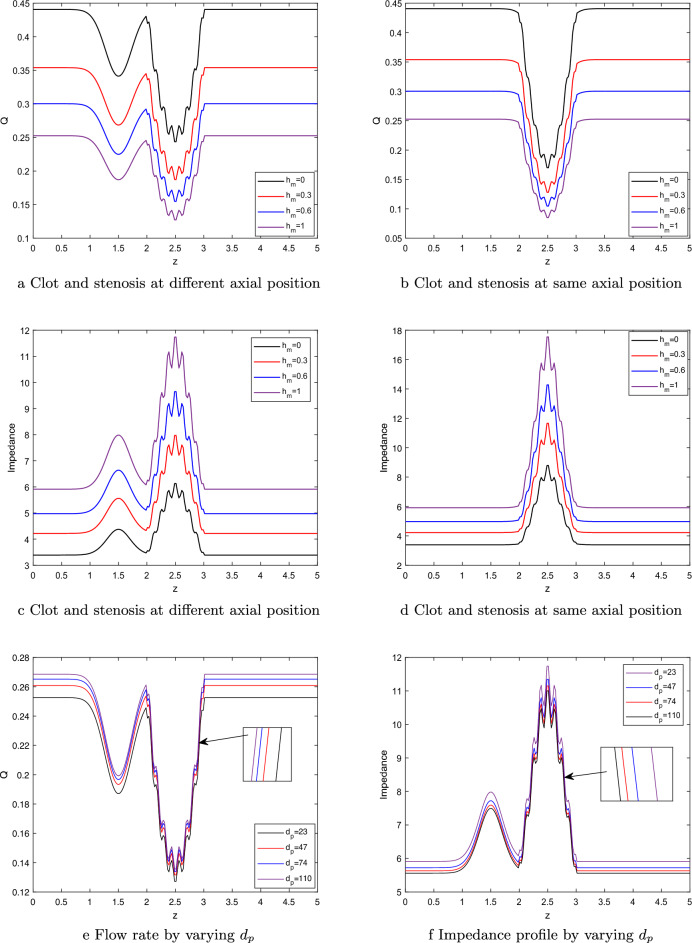
Flow rate by varying (**a**) $h_m$,     (**b**) $h_m$,     Impedance profile by varying (**c**) $h_m$,    (**d**) $h_m$,      (**e**)Flow rate by varying $d_p$,      (**f**) Impedance profile by varying $d_p$.

The present study illustrates the influence of the hematocrit parameter on the flow rate through the utilization of Fig.  7a,b. In Figure 7a, the first scenario is depicted, wherein the clot is positioned on the left side of the stenosis. Conversely, Fig. 7b portrays the second scenario, wherein both the clot’s axial position and location remain consistent. Clearly, from the figures, it can be seen that the flow rate is less in Fig. 7a as compared to Fig. 7b at the axial position $z_1=2$ to $z_1=3$. This has happened due to hindrance observed by the flow from both clot and stenosis simultaneously, while the flow rate in Fig.  7a first reduces due to clot presence and then decreases due to stenosis presence. In both depicted illustrations, a noticeable decrement in the flow rate, accompanied by an enhancement in the hematocrit parameter. The observed phenomenon can be elucidated by the simultaneous increase in fluid viscosity resulting from higher levels of hematocrit concentrations in the blood. Similarly, from the Fig. 7c,d, we can observed that the impedance profile is higher for the case 2 as compared to case 1. In first case, fluid first experience the obstruction due to clot placed at the catheter, then due to stenosis at the arterial wall. In the second case, as the stenosis and clot are located at $z=2.5$ (positioned at same axial position), so the fluid (blood) experience more hindrance due to their combined effect as compared to first case, where the hindrance in fluid path exists independently. In both the figures, the impedance profile shows increasing nature with respect to the hematocrit parameter. The fluid viscosity increases with an enhancement in the hematocrit parameter leading to show declination in the fluid velocity due to hindrance in its path. Figure 7e,f potrayed the flow rate and impedance profile for the distinct nanoparticle size , respectively. Clearly, from the figure, it can be inferred that flow rate increases as the nanoparticle size enhances as observed in the Fig. 7e, while shows the declining nature in the impedance profile as depicted by Fig.  7f. The smaller the size of the nanoparticle then fluid has more viscosity. Thus, increasing the nanoparticle size reduces the fluid viscosity leading to increasing the flow rate profile whereas decreasing impedance profile as less hindrance comes to the fluid path.

### Heat transfer coefficient profile and wall shear stress (WSS)

**Figure 8:**
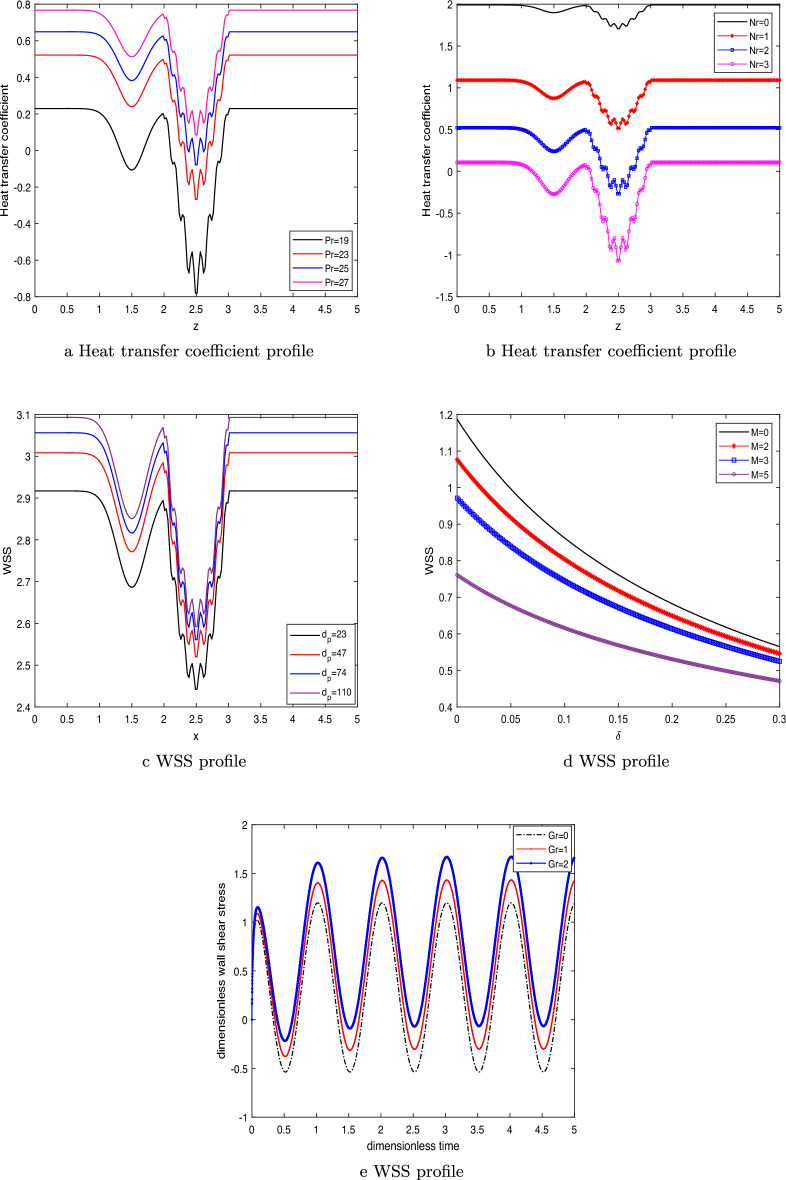
Heat transfer coefficient profile by varying (**a**) *Pr*,    (**b**) *Nr*,      (**c**) WSS by varying $d_p$,     (**d**) WSS by varying stenosis depth $\delta$ and *M* ,     (**e**) WSS by varying *t* and *Gr*.

The profile of the heat transfer coefficient for flow parameters like the Prandtl number and the radiation parameter are depicted in Fig. 8a,b, respectively. It may be noted from the Fig. 8a that the heat transfer profile at axial position $z_1=2.5$ (peak value of stenosis), the change in heat transfer profile is nearly 65% for change in *Pr* from 19 to 23.

While, the enhancement is nearly $200 \%$ for change of *Pr* from 25 to 27. Similarly, we can observed from the graph that near the clot region (clot position shifted left of stenosis) at $z_1=1.5$, the change is nearly $300\%$ for *Pr* ranging from 19 to 23 and nearly $35 \%$ for *Pr* ranging from 25 to 27. Also, we can inferred that the heat transfer efficiency profile rises to a higher level as the value of *Pr* is increased. It may be justified since there has been a drop in the thickness of the thermal boundary layer. The reduced temperature profile is the consequence of the reduction in thermal conductivity. As a result, the coefficient of heat transmission through the wall increases. The decrease in thermal conductivity that can be seen in Fig. 8b is shown to occur when the radiation parameter is increased. The reversal trend is seen as a result of an increase in the thickness of the thermal boundary layer close to the wall as Nr increases. Therefore, Nr brings about a decrease in the heat transmission coefficient. From the figure, we can observed that at the stenosis peak, the change in heat transfer profile is nearly $69\%$ and nearly $300\%$ for change in *Nr* from 0 to 1 and 2 to 3, respectively. While, near the clot region, the change in heat transfer profile is nearly $53\%$ and nearly $200\%$ for change in *Nr* from 0 to 1 and 2 to 3, respectively. The change in percentage value for both the figures can be occur due to amplifying nature of irregular stenosis and clot with change in the parameter values.

Figure 8c shows how the size of the nanoparticles affects the WSS distribution. As the value of parameter $d_p$ grows, a rising trend is seen in the WSS profile. This is because reducing the nanoparticles size has lowered down the fluid’s viscosity, which has increased fluid flow and rendered WSS a growing function of nanoparticle size. When both the stenotic depth and the magnetic field parameter increase, the WSS profile decreases, as seen in Fig. 8d. The fluid’s velocity slows as a result of Lorentz force acting against it. As well as increasing the stenotic depth decreases the fluid velocity as it experiences hindrance in its path with an increase in the size of the stenosis. Grashof number’s influence on the shear stress profile is seen in Fig.  8e. The amplitude develops slowly at first and then oscillates at regular intervals. As Gr increases from 0 to 2, the fluid flow enhances due to the generation of thermo buoyancy force.

### Velocity contour

**Figure 9:**
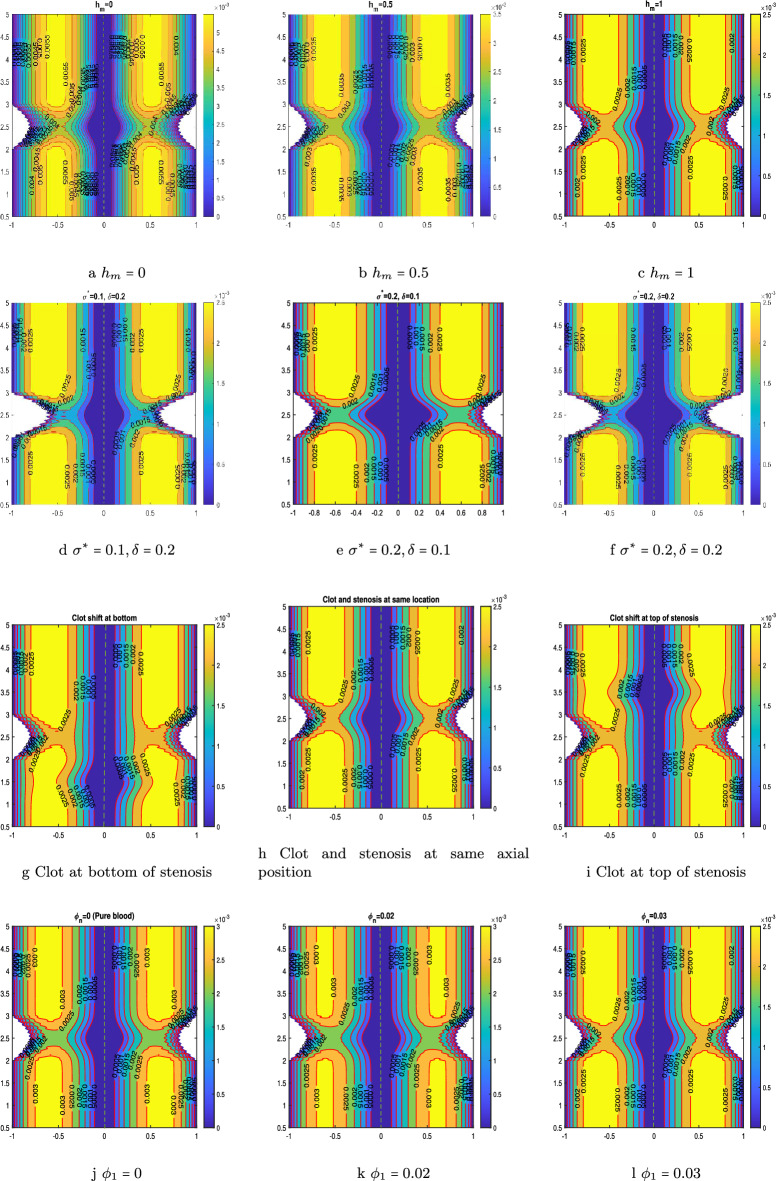
Velocity contour by varying hematocrit parameter, by varying stenosis and clot size, by varying the position of the clot, by varying nanoparticle concentration.

The velocity contour provides a visual depiction of the flow, which may be used to analyse the effect of various parameters on the flow field. Velocity contours display the velocity magnitude at different arterial locations by the series of color-coded regions. The contour for a range of hematocrit values, from $h_m=0$ to $h_m=1$, is portrayed in Fig. 9a–c. The artery section consider here lies in the region of $z=0.5$ to $z=5$. It can be concluded from the figure that the velocity of the fluid reduces from 0.0055 to 0.0025. This declination is justified from the Fig.  9a–c as the magnitude of hematocrit parameter augmented the fluid viscosity also enhances that is depicted by the reduction in the flow rate. Figure 9d-f portrays the role of the size of the stenosis and clot on the hemodynamic flow. The fluid velocity reduces as the size of the stenosis or clot increases as depicted in the figure. If the Fig. 9d, e are compared with Fig. 9f, the reduction in the velocity can be observed near the region occupying the clot and stenosis in the span of $z=2$ to $z=3$.

The hindrance comes in the fluid path can be catastrophic as it reduced the blood flow through artery which is necessary for the basic functions of the body. Thus, it is necessary to address this behavior and proper cure for the disease at a right time. The impact of the clot location on the hemodynamic flow issue is shown in Fig. 9g–i. Although the maximum flow velocity remains the same in all three conditions, but the change in flow velocity in a certain region can be observed from the figure. In Fig. 9g, the position of the clot comes first afterwards, the stenosis. Similarly, in Fig. 9i, the position of the stenosis comes first afterwards the clot, while in Fig. 9h, the stenosis and clot are centered at the same axial position. In Fig. 9h, the stenosis and clot are centered at the same axial position. Compared to the other two situations, the flow velocity is lower in scenario 9h because the clot and stenosis act together to provide a multiplicative effect on the resistance to blood flow. Figure 9j–l depicts the velocity profile for different nanoparticle concentrations from 0 to 0.03. Figure 9j represents the arterial section when no nanoparticle is mixed with the blood, while the other two cases (see 9k,l) are for nanoparticle concentrations 0.02 and 0.03. As the concentration of nanoparticles in the blood increases, the velocity profile decreases. These methods may be beneficial for medical professionals and surgeons to slow the body’s blood circulation.

### Entropy

**Figure 10:**
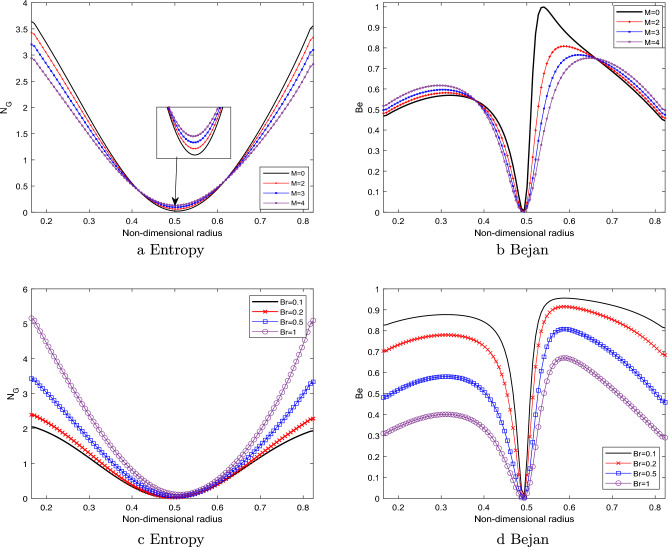
Entropy by varying (**a**) *M*,    Bejan number by varying (**b**) *M*,     Entropy by varying (**c**) *Br*,     Bejan number by varying (**d**) *Br*.

Figure 10a,b depict the effect of magnetic field parameter on the entropy generation $N_G$ and Bejan number *Be*, respectively. The figure demonstrates a pattern in which the entropy initially decreases, followed by an increase, and ultimately reduces again as the magnetic field parameter is enhanced. While, the reversed behavior is observed with the Bejan number profile as depicted in Fig. 10b.

It is noticed from Fig. 10a that the entropy generation profile decreases as the magnetic field parameter enhances near the arterial wall and catheter tube. This has happened due to the fluid friction irreversibility arises from the resistive Lorentz force. As, we move away from the walls near the center of the artery, the heat transfer irreversibility dominates due to presence of strong magnetic field which raises the temperature due to Ohmic heating. Hence, the entropy generation enhances near the center. Figure 10c elaborates the entropy generation profile for different values of the Brinkmann number *Br*. Brinkmann number is the ratio of heat generated due to viscous dissipation and heat transported by the molecular conduction. It is evident from the figure that there is a discernible correlation between the Brinkmann number and the enhancement of the entropy profile. This has happened due to the less prominent effect of viscous dissipation as compared heat transfer by molecular conduction. The substantial amount of heat generated between the layer of the fluid causing an enhancement in the entropy profile. The reverse behavior is observed in the Bejan number profile as depicted in the Fig.  10d. The declination in the Bejan profile can be explained by the fact that the less dominant effect of the molecular conduction as compared to viscous dissipation effect.

## Conclusion

The present mathematical model provides the deep insight into the rheology of blood subject to pathological conditions such as stenosis and thrombosis and further helps scientists and researchers to understand the blood flow characteristics. The reduced form of governing equations are discretized using the Crank–Nicholson method and the relevant profile are computed. The salient findings are delineated as follows:

- The velocity distribution demonstrates an increase with an escalation in the nanoparticles volumetric concentration or the Grashof number, primarily due to the intensified effect of buoyant forces .
- The decrement in the WSS profile is observed with an increment in stenotic depth or the magnetic field parameter *M*.
- The velocity profile exhibits a negative correlation with the magnetic field intensity, while a positive correlation is observed between the velocity profile and the Debyle length parameter.
- Increasing the Brinkmann paramter *Br* enhances the entropy generation profile but shows the reverse trend with the Bejan number.

The current investigation entails the incorporation of aluminium oxide nanoparticles (AlNPs) into the base fluid medium. The nanoscale entities are categorized as porous metallic oxides, which possess significant surface areas and strongly resist chemical and mechanical disturbances. The extensive accessibility of these technologies makes them economically feasible for integration into the field of biomedical applications. In addition, the aluminium nanoparticles (AlNPs) exhibit significant chemical stability even under exposure to abrasive environments. Examining various dimensions and configurations of nanoparticles within the curved artery facilitates researchers in acquiring knowledge pertaining to the customization and production of pharmaceuticals to enhance drug delivery systems’ efficacy. Entropy analysis allows researchers to quantitatively assess the degree of disorder or randomness displayed by flow patterns and evaluate energy dissipation within the system. The present study has primarily focused on standard wall conditions. It is imperative to extend the investigation by considering the permeable wall conditions to advance the research in this domain. To Utilize the magnetic drug targeting to treat stenosed arteries with aneurysms and other pathological conditions. The current study has yet to delve into the complexities of two-phase blood flow modelling. Incorporating the two-phase blood flow model to analyze the fluid flow and heat transfer in a curved tube with time-variant stenosis can significantly broaden the research.

## Data Availability

The datasets used and/or analysed during the current study available from the corresponding author on reasonable request.

## List of symbols

$$u_1=\frac{L_0 \tilde{u}_1 }{\delta ^* U_0}$$: Radial velocity
$U_0$: Reference velocity (ms$^{-1}$)
$$w_1=\frac{\tilde{w}_1}{U_0}$$: Axial velocity
*g*: Acceleration due to gravity (ms$^{-2}$)
$$z_1=\frac{\tilde{z}_1}{L_0}$$: Axial direction
$$r_1=\frac{\tilde{r}_1}{R_0}$$: Radial direction
*T*: Temperature (K)
$$t= \frac{U_0 \tilde{t}}{R_0}$$: Time
$$\tilde{T}_0$$: Reference temperature (K)
$\tilde{C}_0$: Reference concnentration (mol m$^{-3}$)
$\tilde{T}_w$: Temperature at wall (K)
$\tilde{C}_w$: Concentration at wall (mol m$^{-3}$)
*Q*: Volumetric flow rate (m$^{3}$/s)
$R_0$: Radius of normal artery (m)
*e*: Systolic to diastolic pressure ratio
*B*: Uniform magnetic field (T)
$R_c= \frac{\tilde{R}^*}{R_0}$: Radius curvature of artery
$$Nr=\frac{16 \sigma _e \tilde{T}_0^3}{3 \kappa _f\kappa _e}$$: Radiation parameter
$f_p$: Heart pulse frequency
$Gr= \frac{g(\rho \beta)_{f} R_0^2 (\tilde{T}_w-\tilde{T}_0)}{\mu _0 U_0}$: Thermal Grashof number
$Gc= \frac{g(\rho \beta)_{f} R_0^2 (\tilde{C}_w-\tilde{C}_0)}{\mu _0 U_0}$: Solutal Grashof number
$$Re=\frac{U_0\rho _fR_0}{\mu _0}$$: Reynold’s number
$$Ec=\frac{U_0}{c_p(\tilde{T}_w-\tilde{T}_0)}$$: Eckert number
$$Pr=\frac{\mu _0 C_p}{\kappa _f}$$: Prandtl number
$E_1=\frac{R_0}{\sqrt{\mu _0} U_0 } E_0$: Electric field parameter
$p=\frac{R_0^2 \tilde{p}}{\mu _0 U_0 L_0}$: Non dimensional pressure
$q_e=\frac{R_0}{q_m}$: Electro-osmotic parameter
$S_z= \frac{\sigma _f}{\kappa _f} \frac{R_0^2 E_0^2}{(\tilde{T}_w-\tilde{T}_0)}$: Joule heating term
$U_{hs}=\frac{\zeta \epsilon E_0}{\mu _0 U_0}$: Helmholtz–Smoluchowski velocity
$$Sc=\frac{\nu }{D_m}$$: Schmidt number
$M^2=\frac{\sigma _f B_0^2 R_0^2}{\mu _0}$: Magnetic field parameter

## Greek letters

$$\sigma $$: Electrical conductivity (S/m)
$$ (C_{p})_{nf}$$: Specific heat at constant pressure
$\rho _{nf}$: Density of nano-fluid (Kg/m$^{3}$)
*e*: Systolic to diastolic pressure ratio
$$\delta ^* = \frac{\delta }{R_0}$$: Stenosis depth
$$\kappa _f$$: Thermal conductivity (W/(m K))
$\tau _w$: Wall shear stress (Pa)
$$\omega _p$$: Circular frequency
$$\lambda $$: Impedance ($$\Omega $$(Ohm))
$\beta$: Thermal expansion coefficient (K$^{-1}$)
$\mu _f$: Blood’s viscosity (Pa s)
$\xi =\frac{R_b \rho _f R_0^2}{\mu _0}$: Chemical reaction parameter
$\theta _1=\frac{\tilde{T}-\tilde{T}_0}{\tilde{T}_w-\tilde{T}_0}$: Non-dimensional temperature
$\phi _1=\frac{\tilde{C}-\tilde{C}_0}{\tilde{C}_w-\tilde{C}_0}$: Non-dimensional temperature
$$\Omega =\frac{\tilde{T}}{(\tilde{T}_w-\tilde{T}_0)}$$: Temperature difference
$\mu _0$: Reference viscosity (Pa s)
$$\Lambda =\frac{\tilde{C}}{(\tilde{C}_w-\tilde{C}_0)}$$: Mass difference
